# Integrated Multi-Omics and Interactome Analysis of CDK8 Inhibition Reveals Erythroid Differentiation Programs and Therapeutic Synergy with BET Blockade in AML

**DOI:** 10.3390/cells15151414

**Published:** 2026-08-04

**Authors:** Malgorzata Statkiewicz, Izabela Rumienczyk, Urszula Pakulska, Marta Obacz, Maria Kulecka, Jarosław Cendrowski, Magdalena Cubulska-Lubak, Ewelina Kaniuga, Zuzanna Sandowska-Markiewicz, Wioletta Slusarczyk-Kacprzyk, Krzysztof Goryca, Tymon Rubel, Magdalena Bakun, Bianka Swiderska, Kamila Kruczkowska-Tarantowicz, Piotr Rzepecki, Jolanta Korsak, Krystyna Kyc-Wachowiak, Anna Polak, Przemyslaw Juszczynski, Milena Mazan, Tomasz Rzymski, Jerzy Ostrowski, Michal Mikula

**Affiliations:** 1 Laboratory of Cancer Metabolism, Department of Experimental Oncology, Maria Sklodowska-Curie National Research Institute of Oncology, 02-781 Warsaw, Polandmkulecka@cmkp.edu.pl (M.K.); jaroslaw.cendrowski@nio.gov.pl (J.C.); jerzy.ostrowski@nio.gov.pl (J.O.); 2 Ryvu Therapeutics, 30-394 Krakow, Poland; usieranska@gmail.com (U.P.); marta.obacz@ryvu.com (M.O.);; 3 Department of Gastroenterology, Hepatology and Clinical Oncology, Centre of Postgraduate Medical Education, 02-781 Warsaw, Poland; 4 Laboratory of Preclinical Research, Department of Experimental Oncology, Maria Sklodowska-Curie National Research Institute of Oncology, 02-781 Warsaw, Poland; magdalena.cybulska-lubak@nio.gov.pl (M.C.-L.); ewelina.kaniuga@nio.gov.pl (E.K.); zuzanna.sandowska-markiewicz@nio.gov.pl (Z.S.-M.); 5 Department of Medical Physics, Maria Sklodowska-Curie National Research Institute of Oncology, 02-781 Warsaw, Poland; wioletta.slusarczyk-kacprzyk@nio.gov.pl; 6 Genomics Core Facility, Centre of New Technologies, University of Warsaw, 02-097 Warsaw, Poland; 7 Institute of Radioelectronics and Multimedia Technology, Warsaw University of Technology, 00-661 Warsaw, Poland; tymon.rubel@pw.edu.pl; 8 Mass Spectrometry Laboratory, Institute of Biochemistry and Biophysics, Polish Academy of Sciences, 02-106 Warsaw, Poland; bakunm@ibb.waw.pl (M.B.); bianka.swiderska@gmail.com (B.S.); 9 Department of Internal Medicine and Hematology, Military Institute of Medicine-National Research Institute, 04-141 Warsaw, Poland; kkruczkowska@wim.mil.pl (K.K.-T.); przepecki@wim.mil.pl (P.R.); 10 Department of Clinical Transfusion Medicine, Military Institute of Medicine-National Research Institute, 04-141 Warsaw, Poland; jkorsak@wim.mil.pl (J.K.); wachowiak1@op.pl (K.K.-W.); 11 Department of Experimental Hematology, Institute of Hematology and Transfusion Medicine, 04-141 Warsaw, Poland; apolak@ihit.waw.pl (A.P.); pjuszczynski@ihit.waw.pl (P.J.)

**Keywords:** leukaemic stem cells, CDK8 inhibitor, protein–protein interactome, chromatin remodelling, BET-CDK8 synergy

## Abstract

**Highlights:**

**What are the main findings?**

- CDK8 inhibition reduced STAT5 S726/731 phosphorylation and promoted differentiation-associated changes in an LSC-enriched TEX cell line AML model.
- CDK8–BET co-inhibition showed context-dependent synergy in AML cell lines and PDX-derived models.

**What are the implications of the main findings?**

- CDK8 supports transcriptional and metabolic programs associated with immature AML states.
- CDK8–BET co-inhibition merits biomarker-guided preclinical evaluation.

**Abstract:**

Acute myeloid leukaemia (AML) is a therapeutically challenging malignancy driven by the self-renewal, quiescence, and therapy resistance of leukaemic stem cells (LSCs). CDK8, a kinase component of the Mediator complex, regulates oncogenic transcription, and the selective CDK8/CDK19 inhibitor RVU120 (Romaciclib) targets AML cells with CD34^+^/pSTAT5-high LSC-like characteristics; however, the epigenetic and transcriptional consequences of CDK8 blockade and actionable combinatorial strategies remain incompletely defined. Using the TEX cell line, an LSC-enriched surrogate model, we performed time-resolved RNA-seq, whole-proteome and phosphoproteomics mass spectrometry (MS), and CUT&Tag chromatin profiling following treatment with RVU120 and CCT251921. CDK8 protein–protein interactions were mapped by co-immunoprecipitation MS across five AML models, and synergy with Pelabresib (BET inhibitor) or CB6644 (RUVBL1/2 inhibitor) was assessed by high-content screening in three cell lines and three patient-derived xenograft (PDX) models. Both inhibitors suppressed STAT5 phosphorylation, induced loss of the CD34^+^/CD38^−^ LSC-enriched phenotype, and drove erythromegakaryocytic differentiation. Transcriptomic and proteomic responses were concordant, and CDK8 inhibition triggered widespread enhancer activation with redistribution of RNAP2, BRD3, and NFRKB. CDK8 combined with Pelabresib acted synergistically in MOLM-16 cells and two of three PDX models. These findings identify CDK8 as a transcriptional node of LSC-associated programs and provide a mechanistic rationale for combined CDK8-BET inhibition in molecularly defined AML subsets, which will require validation in functional LSC assays and primary specimens.

## 1. Introduction

Acute myeloid leukaemia (AML) continues to pose significant challenges in clinical treatment, despite progress with targeted and traditional therapies. One of the main obstacles to achieving long-lasting remission is the presence of leukaemic stem cells (LSCs), a primitive group of progenitor cells that drive the disease through their ability to self-renew, remain quiescent within protective microenvironmental niches, and resist differentiation [1,2]. Unlike the bulk of leukemic blasts, LSCs often display primitive surface markers, such as CD34, and exhibit elevated activity in pathways that support stemness, including STAT5 activation [3]. A key goal of AML therapy is to selectively eliminate LSCs while sparing normal haematopoietic stem and progenitor cells (HSPCs), since LSCs are believed to be the main source of relapse and treatment resistance in most patients [4]. Recent insights from functional genomics and chemical biology have highlighted the Mediator kinase module, especially cyclin-dependent kinase 8 (CDK8), as a crucial transcriptional regulator in AML. CDK8, along with CDK19, Cyclin C, and MED12, forms the core of this complex and plays an important role in controlling both cancer-related transcription and normal blood cell development [5,6]. It functions as a versatile kinase that influences RNA polymerase II (RNAP2) during transcription elongation through the Mediator complex, while also phosphorylating key substrates involved in chromatin organisation and mRNA processing [5].

Over the past two decades, developments in cancer biology have underscored the importance of multi-omics approaches for elucidating kinase functions and identifying promising therapeutic targets. In particular, phosphoproteomics enables the direct detection of kinase substrates and their proximal neighbours, often uncovering unexpected cellular targets that can shed light on downstream signalling pathways [7]. For CDK8, which functions within the Mediator complex but may also act on a wider range of substrates involved in chromatin and RNA processing, phosphoproteomic profiling can help determine which cellular processes are directly influenced by CDK8 kinase activity versus those affected indirectly through changes in transcription [8,9]. Additionally, time-resolved multi-omics techniques, sampling cells at different times after treatment, shed light on the sequence of molecular events, allowing us to differentiate early effects dependent on kinase activity from later responses driven by transcriptional changes [10].

Beyond identifying direct substrates, a complementary approach to understanding CDK8 function involves systematic mapping of its protein–protein interaction (PPI) networks. The Mediator complex is composed of more than 30 subunits organised into distinct functional modules, and CDK8 operates within a densely connected subnetwork that includes cofactors, RNA-processing machinery, and chromatin regulators, including BET proteins [11]. BET proteins, particularly BRD2, BRD3, and BRD4, have emerged as central hubs in the transcriptional control of cancer cells, acting as “readers” of acetylated chromatin through their bromodomains [12]. Of them, BRD4 facilitates the recruitment of the positive transcription elongation factor complex (P-TEFb), which contains CDK9 and Cyclin T, to acetylated super-enhancers that govern oncogenic transcription factors such as MYC and BCL2 [13]. BET inhibitors, which competitively displace BET proteins from acetylated chromatin, have demonstrated preclinical and clinical activity across multiple cancer types and are currently being evaluated in early-phase AML trials [14]. In AML, both CDK8 and BET proteins converge on enhancer-driven transcriptional programs by integrating acetylation-dependent chromatin recruitment with Mediator-mediated signal-responsive transcriptional amplification, thereby controlling oncogenic and lineage-specific gene expression [15].

Our previous work has established that selective CDK8/CDK19 inhibition with RVU120 (Romaciclib; SEL120-34A) suppresses the growth of AML cell lines with LSC-like characteristics, including those expressing high levels of CD34 and elevated Ser726 phosphorylation of STAT5 (pSTAT5) [6]. Currently, RVU120 is being tested as monotherapy in stage II clinical trials for Myelodysplastic Syndrome (MDS) and myelofibrosis, as well as in combination with JAK inhibitors and venetoclax for myelofibrosis and Relapsed/Refractory (R/R) AML, respectively. Despite these advances, key questions persist regarding the temporal dynamics of CDK8 inhibitors’ effects on LSC transcriptional networks and their synergy with conventional therapies. Recent work demonstrates that RVU120 synergises with venetoclax, hinting at combinatorial potential [16]. However, it remains unresolved whether RVU120’s anti-LSC activity derives from direct STAT5 inhibition or broader disruption of Mediator or other chromatin-associated complexes.

To further understand the molecular effects of CDK8 inhibition in LSCs, we undertook an integrated experimental approach combining multi-omics profiling, proteomic CDK8 interactome mapping, functional assays, and pharmacologic combination studies. Using TEX cells, an LSC-like model, we investigated the effects of CDK8 inhibition with two structurally distinct CDK8 inhibitors, RVU120 and CCT251921, on LSC properties, transcriptional and epigenetic programs, and PPIs. Our specific objectives were: (i) to confirm that CDK8 inhibition rapidly suppresses STAT5 phosphorylation and triggers differentiation in LSC-like cells; (ii) to employ integrated multi-omics analyses to define the temporal sequence and molecular coherence of CDK8-dependent transcriptional reprogramming; (iii) to perform survey of the CDK8 interactome and identify high-confidence, conserved interactors across multiple AML models; (iv) to identify the most abundant and druggable CDK8 interacting proteins suitable for combination therapy, and (v) to evaluate the translational potential of combinational CDK8 inhibition using multiple AML cell lines and patient-derived xenograft (PDX) models. Collectively, our experimental results, addressing the above objectives, position CDK8 as a central regulatory node in LSC models maintenance and define a rational combination therapy approach for a clinically actionable subset of AML patients.

## 2. Materials and Methods

### 2.1. Chemicals

RVU120, CCT251921 and Pelabresib were provided by Ryvu (Krakow, Poland). CB6644 (Cat. No. SML3892) was purchased from Merck (Rahway, NJ, USA). All chemical compounds were dissolved in Dimethyl Sulfoxide (DMSO) to prepare a 10 mM stock solution and were stored at −80 °C.

### 2.2. Cell Culture

AML cell lines were obtained from ATCC; TEX cells were provided by Dr. John Dick. Cells were cultured at 37 °C with 5% CO_2_. TEX and KG-1 cells were maintained in supplemented IMDM containing FBS, SCF and IL-3, whereas MOLM-13, MOLM-14, MOLM-16 and MV4-11 were cultured in RPMI-1640 with 20% FBS. Cell-line identity and mycoplasma-free status were confirmed. Details on culture conditions are provided in the Supplementary Methods file.

### 2.3. AML PDX Development

Patient-derived AML cells were obtained from the Institute of Hematology and Transfusion Medicine and the Military Institute of Medicine-National Research Institute. AMLX4 cells were purchased from Proxe (Boston, MA, USA). The cells were injected via the tail vein into 8-week-old female NSG-SGM3/J mice 24 h after 2.25 Gy of whole-body irradiation. Following cells isolation from mouse bone marrow, the engraftment of human CD45 cells was confirmed using FACS. The established PDXs AML cells were stored long-term in liquid nitrogen.

### 2.4. PDX-Derived AML Cells Culture and Treatment

PDX-derived AML cells were isolated from mouse bone marrow and cryopreserved long-term in liquid nitrogen. For in vitro experiments, frozen cells were thawed and allowed to recover for 24 h in a specially enriched medium consisting of IMDM supplemented with BIT (15%), β-mercaptoethanol (0.1 mM), 1:100 Penicillin/Streptomycin, 1 mM sodium pyruvate, and a cytokine/inhibitor cocktail including SR1 (500 nM), UM729 (500 nM), SCF (100 ng/mL), G-CSF (20 ng/mL), FLT3-L (50 ng/mL), and IL-3 (20 ng/mL). After recovery, cells were maintained in suspension culture under standard conditions (37 °C, 5% CO_2_) and used for downstream synergy assays.

### 2.5. Flow Cytometry

TEX cells were cultured with EPO and treated with 0.5 µM RVU120 for up to 14 days. At the indicated time points, cells were Fc-blocked and stained for CD34, CD38, CD41 and CD71, with matched isotype controls and a viability dye. Samples were analysed using a BD LSR Fortessa cytometer (BD Biosciences, San Jose, CA, USA) and FlowJo v10.8.1. More details on the conditions are provided in the Supplementary Methods file.

### 2.6. RNA Isolation and Sequencing

Total RNA was isolated from 1 million TEX cells using the DirectZol kit (Zymo Research, Irvine, CA, USA). All samples were DNase-treated and eluted with 50 µL of water. The quality and quantity of isolated RNA were analysed using the 2100 Bioanalyzer System and Eukaryote Total RNA Nano Series II Kit (Agilent, Santa Clara, CA, USA). RNA-Seq as a Coding Transcriptome Sequencing (CTS) service was performed by CeGaT (Tübingen, Germany) (≥1 µg of RNA with a concentration ≥ 40 ng/µL in at least 25 µL total volume).

### 2.7. RNA-Seq Data Processing

Demultiplexing of the sequencing reads was performed with Illumina (San Diego, CA, USA) bcl2fastq (2.20). Adapters were trimmed with Skewer (version 0.2.2) [17]. The quality of the FASTQ files was analysed with FastQC (version 0.11.5-cegat). The resulting fastq files were mapped to the reference genome (hg38) using the STAR aligner [18] version 2.7.10a with default parameters. The read assignments per gene were counted using htseq-count [19] (version 0.11.1), using default counting mode and reverse string assignment to genes.

### 2.8. Protein Immunoprecipitation for Western Blotting

TEX cells (5 × 10^6^ cells per sample) were lysed in inhibitor-supplemented immunoprecipitation (IP) buffer, sonicated and clarified. Lysates were incubated overnight at 4 °C with 5 µg of NFRKB, BRD3 or CDK8 antibody, followed by Protein A Dynabeads for 4 h. Immunoprecipitated proteins were eluted in Laemmli buffer by heating at 99 °C for 5 min and stored at −20 °C. Detailed protocol is provided in the Supplementary Methods file.

### 2.9. Western Blotting

Cells were lysed in inhibitor-supplemented RIPA buffer, and protein concentrations were determined by Bradford assay. Equal amounts of protein were separated by SDS-PAGE and transferred to PVDF membranes. Membranes were probed for NFRKB, BRD3, CDK8, STAT5, phospho-STAT5, actin and vinculin, followed by HRP-conjugated secondary antibodies and ChemiDoc (Biorad, Hercules, CA, USA) visualisation. Details on protocol and antibodies are provided in the Supplementary Methods file.

### 2.10. Proteins Digestion

Cell pellets were lysed in TFE/TCEP/TEAB buffer by sonication and heating. Cysteines were blocked with MMTS, and proteins were digested overnight with trypsin. Peptides were purified using Oasis HLB columns and quantified. Equal peptide amounts formed an internal standard for inter-set normalisation. Samples and the standard were labelled with TMTpro 18-plex reagents (Cat. No. A52045, Thermo Fisher Scientific, Waltham, MA, USA), pooled, desalted and dried. Detailed protocol is provided in the Supplementary Methods file.

### 2.11. Reversed-Phase Peptide Fractionation at High pH

Each TMT set (540 µg) was fractionated by high-pH reversed-phase UPLC on an XBridge BEH C18 column using a 64 min water/ACN gradient containing ammonium hydroxide (0.8 mL/min). Sixty one-minute fractions were concatenated into 30 samples for LC-MS analysis (2 µg each). Remaining peptides were pooled, desalted, and subjected to phosphopeptide enrichment. Detailed protocol is provided in the Supplementary Methods file.

### 2.12. Phosphopeptide Enrichment with Ti-IMAC

Phosphopeptide enrichment was performed using MagReSyn Ti-IMAC beads (Resyn Biosciences, Gauteng, South Africa) according to the protocol provided in the Supplementary Methods file.

### 2.13. Protein Immunoprecipitation for Mass Spectrometry

Protein A Dynabeads were coupled to 5 µg rabbit IgG control or anti-CDK8 antibody (Bethyl, A302-500A) and crosslinked with 5 mM BS3. Lysates from 5 × 10^6^ AML cell-line or PDX cells were prepared in inhibitor-supplemented IP buffer, sonicated, and clarified. Lysates were sequentially incubated with IgG- and CDK8-coupled beads. Complexes were washed, eluted with 200 mM formic acid, and stored at −80 °C for MS analysis. Detailed protocol is provided in the Supplementary Methods file.

### 2.14. Mass Spectrometry

Co-IP, global TMT and phospho-enriched TMT samples were analysed using an Evosep One (Evosep Biosystems, Odense C, Denmark) coupled to an Orbitrap Exploris 480 (Thermo Fisher Scientific, Waltham, MA, USA). Global samples used a 44 min gradient, whereas phosphopeptide and co-IP samples used an 88 min gradient. Peptides were analysed by positive-mode data-dependent acquisition with HCD fragmentation. Detailed protocol is provided in the Supplementary Methods file.

### 2.15. Mass Spectrometry Data Analysis

Proteins and peptides were identified using MaxQuant/Andromeda against the human SwissProt database at 1% FDR. TMT reporter-ion intensities were normalised using internal reference scaling and LOESS. Co-IP data were quantified by spectral counting and the APEX index. Phosphoproteomic analysis included singly phosphorylated peptides quantified across all replicates, with site-localisation probability ≥ 0.90 and normalisation to corresponding protein abundance. Differential proteins and phosphosites were identified using resampling-based statistics with Benjamini–Hochberg-adjusted *p* < 0.05 and fold change ≥ 1.25. Detailed protocol is provided in the Supplementary Methods file.

### 2.16. CUT&Tag Libraries Preparation and Sequencings

TEX cells were treated with 0.5 µM RVU120 or CCT251921 for 24 or 72 h. CUT&Tag was performed using 300,000 cells per antibody and a CUT&Tag-IT Assay Kit (Active Motif, Carlsbad, CA, USA). Antibodies targeted H3K27ac, H3K4me1, H3K4me3, RNAP2, MLL4, CDK8, BRD3 and NFRKB. Libraries were normalised, pooled and sequenced as 75 bp paired-end reads using a NovaSeq 6000 (Illumina, San Diego, CA, USA). Detailed protocol is provided in the Supplementary Methods file.

### 2.17. CUT&Tag Data Analyses

Reads were quality-filtered and adapter-trimmed with Trimmomatic, aligned to the GRCh38 genome with Bowtie2 and deduplicated using GATK MarkDuplicates. RPKM-normalised coverage tracks were generated with bamCoverage, and peaks were identified using MACS2. Genomic and functional annotations were performed using ChIPpeakAnno (version 3.36.1) and ReactomePA (version 1.46.0), respectively, and binding profiles were generated with deepTools (version 3.5.1). Detailed protocol is provided in the Supplementary Methods file.

### 2.18. High-Content Microscopy Analyses and Synergy Testing

AML cell lines (KG-1, MOLM-13 and MOLM-16) and PDX-derived cells (AMLX4, AMLX12 and AMLX53) were treated in triplicate with CDK8 inhibitors alone or combined with pelabresib or CB6644. Fixed, Hoechst-stained cells were imaged using the Opera Phenix system and quantified with Harmony 4.9. DNA replication in MOLM-16 was assessed by EdU incorporation. Drug interactions based on live-cell counts were evaluated using SynergyFinder 3.0. Detailed protocol is provided in the Supplementary Methods file.

## 3. Results

### 3.1. RVU120 Inhibits STAT5 Phosphorylation at S726/731 and Triggers LSC-like TEX Cell Line Differentiation

**Figure 1:**
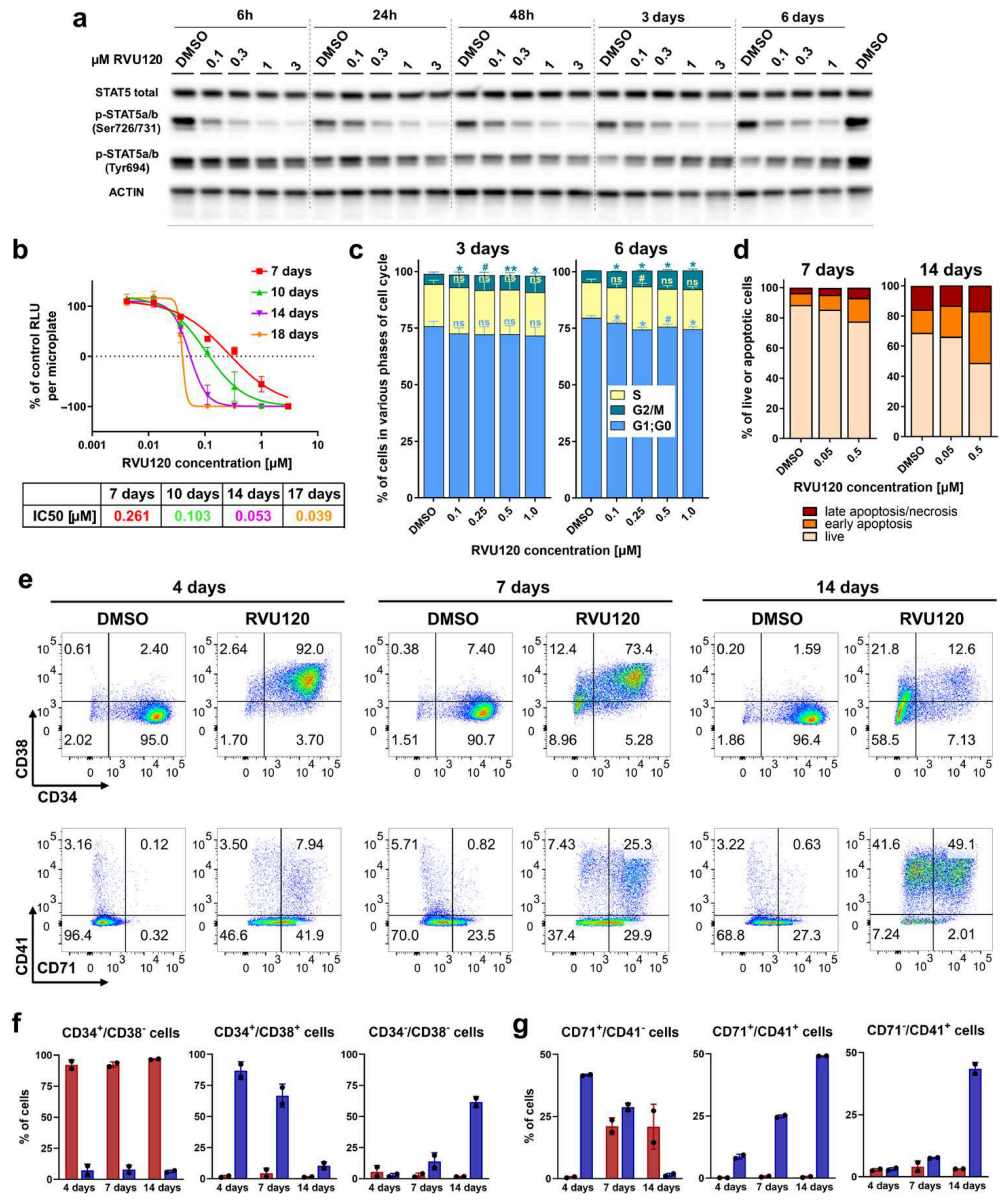
RVU120 inhibits STAT5 phosphorylation at S726 and triggers LSC-like TEX cell line differentiation. (**a**) STAT5 expression and p-STAT5 (S726/S731)/p-STAT5 (Y694) abundance evaluation in TEX cells treated with the indicated concentrations of RVU120 for 6, 24, 48, 72 and 144 h. Actin and Vinculin were used as loading controls. Representative Western blots are shown (n = 2 and n = 3 for 24, 72 and 144 h). (**b**) Long-term viability of TEX cells treated with increasing concentrations of RVU120 for 7, 10, 14, and 18 days. Data are mean ± SD of technical triplicates from a representative experiment (n = 3). A representative graph is shown. (**c**) Cell cycle analysis based on flow cytometric detection of incorporated BrdU and DAPI staining intensity in TEX cells treated for 3 or 6 days with DMSO or RVU120 at the indicated concentrations. Mean values (±SEM) of the cell percentage in each cell cycle phase (S, G2/M or G1; G0) compared statistically to the results for DMSO-treated cells (n = 3, for 3 days and n = 4 for 6 days). The analysis includes the representative flow cytometry experiment shown in Figure S1. (**d**) Representative results of flow cytometric cell death assessment in TEX cells stained with Annexin V and 7-AAD after treatment with DMSO or RVU120 at indicated concentrations for 7 or 14 days. The graphs show the percentage of live, apoptotic or dead cells (n = 1), which is also visualised in the flow cytometry scatter plots in Figure S1. (**e**) Representative flow cytometry immunophenotyping of TEX cells treated with 0.5 µM RVU120 for 4, 7 and 14 days. Scatter plots show the expression of surface markers: CD34, CD38, CD71 and CD41 at the indicated time points. (**f**,**g**) Graphs showing percentages of cells in indicated populations identified by flow cytometry analysis (presented in (**e**)), upon treatment with DMSO or 0.5 µM RVU120 (n = 2 +/− SEM). *p*-values of statistical analysis in (**c**) are highlighted as: ns (*p* > 0.1), **#** (*p* < 0.1), * (*p* < 0.05) or ** (*p* < 0.01).

We previously showed that the growth of AML cell lines with LSC-like characteristics, including CD34^+^ surface antigens and increased pS726/731 STAT5, can be potently inhibited by RVU120 both in vitro and in vivo [6]. In this study, we used the TEX cell line as an LSC-enriched surrogate model for further functional studies. This line was derived from human CD34^+^-enriched Linneg cord blood cells through an engineered disruption of differentiation and self-renewal due to expression of the TLS-ERG oncogene [20]; we note that no cell line constitutes a bona fide LSC, and functional stemness of TEX cells (e.g., serial transplantation) was not assessed here. A time-course treatment of TEX cells with RVU120 resulted in a strong decrease in STAT5 Ser726/731 phosphorylation, observed at 6 h of treatment and sustained through subsequent time points (Figure 1a). Consistent with our findings in other cell lines, we observed that RVU120 impaired TEX cell growth in a dose-dependent and treatment time-dependent manner, with IC_50_ values decreasing from 0.261 to 0.039 between 7-day and 14-day treatments (Figure 1b).

To test whether TEX cell growth inhibition by RVU120 is due to cell cycle arrest or to induced cell death, we performed flow cytometry experiments. Analysing DNA replication by BrdU incorporation assay and total DNA content by DAPI staining, we observed that 3- or 6-day RVU120 treatment caused a modest increase in the percentage of cells in the G2/M phase, indicating a slight delay in mitotic progression (Figure S1a and Figure 1c). On the other hand, by staining cells with Annexin V and 7-AAD cell death markers, we observed a gradual increase in apoptosis between 7 and 14 days of treatment (Figure S1b and Figure 1d). Hence, we concluded that the effect of RVU120 on TEX cell growth was not due to cell cycle inhibition but rather to increased apoptotic death. To assess whether RVU120 could affect the LSC properties of TEX cells, we measured surface levels of CD34 and CD38, haematopoietic progenitor markers, by flow cytometry. Whereas the majority of DMSO-treated TEX cells displayed the CD34^+^/CD38 LSC-enriched immunophenotype, consistent with their primitive myeloid progenitor state, 4-day RVU120 treatment led to a marked upregulation of CD38 expression, with more than 90% of cells exhibiting a CD34^+^/CD38^+^ committed myeloid progenitor phenotype (Figure 1e,f). Prolonged exposure to RVU120 (for 7 and 14 days) resulted in a gradual loss of both CD34 and CD38 progenitor markers (Figure 1e,f), indicating myeloid lineage maturation. The LSC properties of the TEX cell line may be due to an early block of differentiation by ERG oncogenic signalling, known to primarily suppress erythroid or megakaryocytic differentiation [21,22]. Hence, we assessed by flow cytometry the effect of RVU120 on the surface levels of CD71 and CD41, markers of erythroid and megakaryocytic lineage maturation. Throughout the course of RVU120 treatment, we observed an early (4 days) accumulation of CD71^+^/CD41^−^ cells, followed by a gradual enrichment in CD71^+^/CD41^+^ bipotent precursors and a late (14 days) accumulation of CD71^−^/CD41^+^ cells (Figure 1e,g), suggesting a gradual mixed maturation toward both lineages. [23]. Hence, targeting STAT5 phosphorylation in LSC-like model TEX cells, using RVU120, coincided with loss of the CD34^+^/CD38^−^ LSC-enriched immunophenotype and a gradual differentiation and maturation of TEX cells. These immunophenotypic and differentiation readouts indicate that the TEX cells are a suitable surrogate model for studying RVU120’s mode of action on LSC-associated programs.

### 3.2. CDK8 Inhibition Drives Time-Dependent Erythroid-like Differentiation Programs in TEX Cells Revealed by Integrated Multi-Omics Analyses

**Figure 2:**
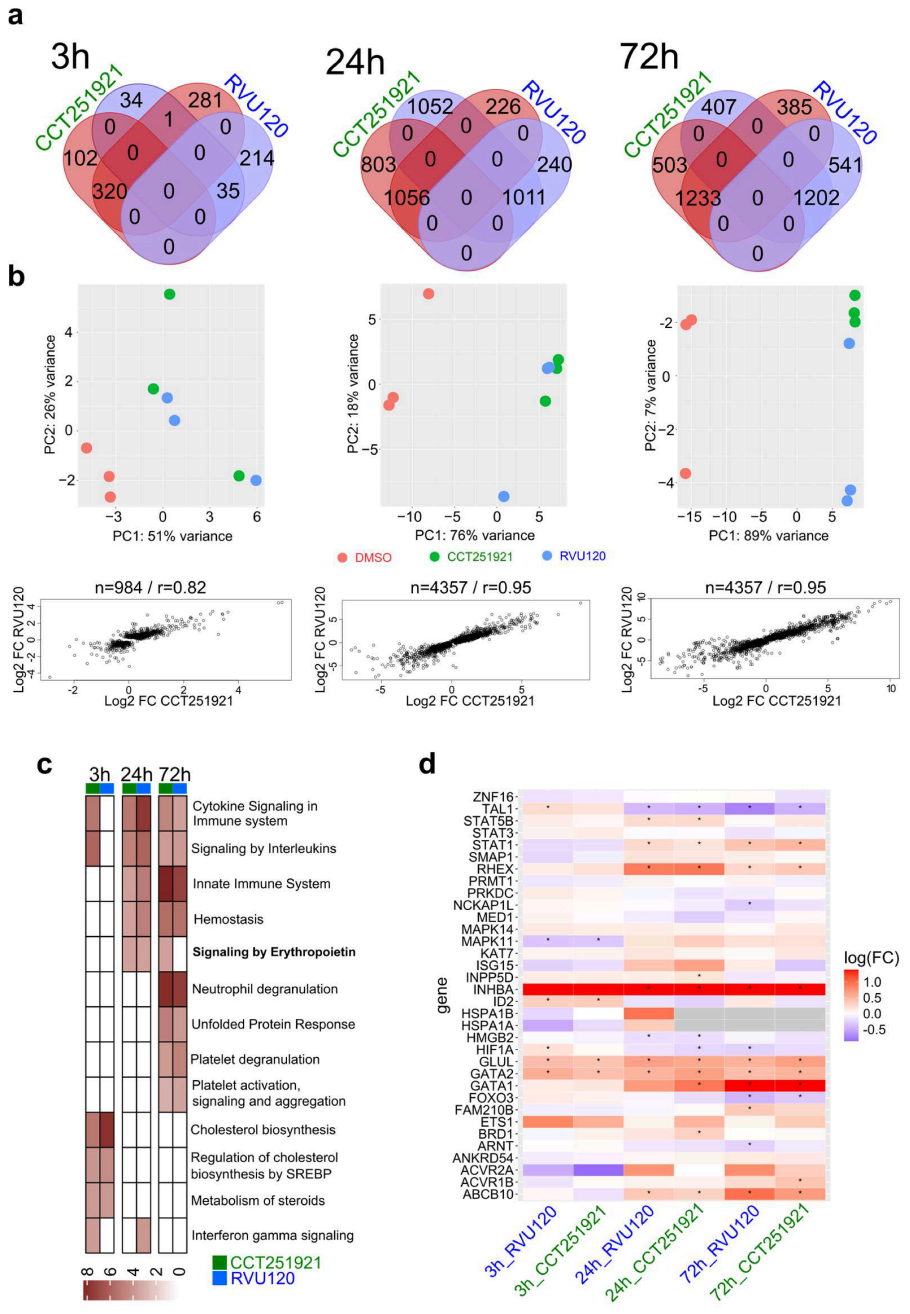
Transcriptomic profiling and functional changes in the TEX cell line following CDK8 inhibition. (**a**) Venn diagrams with overlapping numbers of differentially expressed genes (DEGs) at 3 h, 24 h and 74 h following the RVU120 and CCT251921 treatment in the TEX cell line. Red and blue shading denote up- and downregulated DEGs, respectively. (**b**) Principal component analysis (upper panel) and correlation coefficients (lower panel) of the RNA-seq dataset for the TEX cell line treated with RVU120 or CCT251921 for 3, 24, and 72 h indicate highly consistent transcriptomic responses to both compounds. (**c**) Significantly altered (adj. *p*-value < 0.05) recurrent Reactome terms for the upregulated DEGs after the RVU120 and CCT251921 treatment. The *p*-value is shown on the PHRED scale. (**d**) Heatmap of genes playing an important role in erythropoiesis. The expression values were extracted from the RNA-Seq dataset. DEGs with adj. *p*-values < 0.05 are highlighted with an asterisk (*).

We reasoned that the long-term effects of CDK8 inhibition on LSC survival, differentiation and maturation (observed between 3 and 18 days of inhibitor treatment in Figure 1b–g) must be downstream consequences of earlier mechanistic events, such as altered STAT5 phosphorylation. To investigate the early molecular effects of CDK8/19 inhibition on LSC-like cell line characteristics, we conducted transcriptomic, proteomic, and phosphoproteomic analyses at 3, 24, and 72 h post-inhibitor treatment. To reduce the influence of compound-specific off-target activities and to enrich for on-target CDK8/CDK19 effects, in addition to RVU120, we used a second, structurally distinct CDK8/19 inhibitor, CCT251921 [24], which in the TEX cell line inhibited STAT5 phosphorylation (Figure S2a) and cell growth in a dose- and time-dependent manner (Figure S2b). Transcriptomic analyses identified 850/492, 2544/3956, and 3372/3361 differentially expressed genes (DEGs) at an adjusted *p*-value ≤ 0.05, respectively, for RVU120/CCT251921 at the indicated time points (Figure 2a; Table S1).

Additionally, the survey showed high consistency in the transcriptomic responses to both compounds, as indicated by sample clustering in Principal Component Analysis (PCA) and significant DEGs Spearman correlations of 0.82, 0.95, and 0.95 at the 3, 24, and 72 h time points, respectively (Figure 2b). The DEGs identified by RNA-Seq for a specific experimental condition were then analysed for functional enrichment using the Reactome database to identify the biological processes, molecular functions, and pathways associated with the observed transcriptional changes. The significant pathways were assigned only to overexpressed DEGs (Table S2). For both CDK8 inhibitors, the functional analysis indicated potential early metabolic priming, with activation of cholesterol biosynthesis, followed by a steadily intensifying inflammatory response and ultimately, a commitment to the erythroid lineage. This was exemplified by activation of the Erythropoietin Signalling pathway, including increased GATA1/2 gene expression, the master regulators of erythroid differentiation (Figure 2c,d). We validated by Western blotting that 72 h treatment of TEX cells with RVU120 or CCT251921 resulted in increased GATA1 protein levels (Figure S2a). We note that both compounds also inhibit the CDK8 paralog CDK19 [6,24], and that potential Mediator-independent or off-target contributions cannot be fully excluded. However, the high concordance between two chemically unrelated inhibitors argues that the shared responses are predominantly on-target.

**Figure 3:**
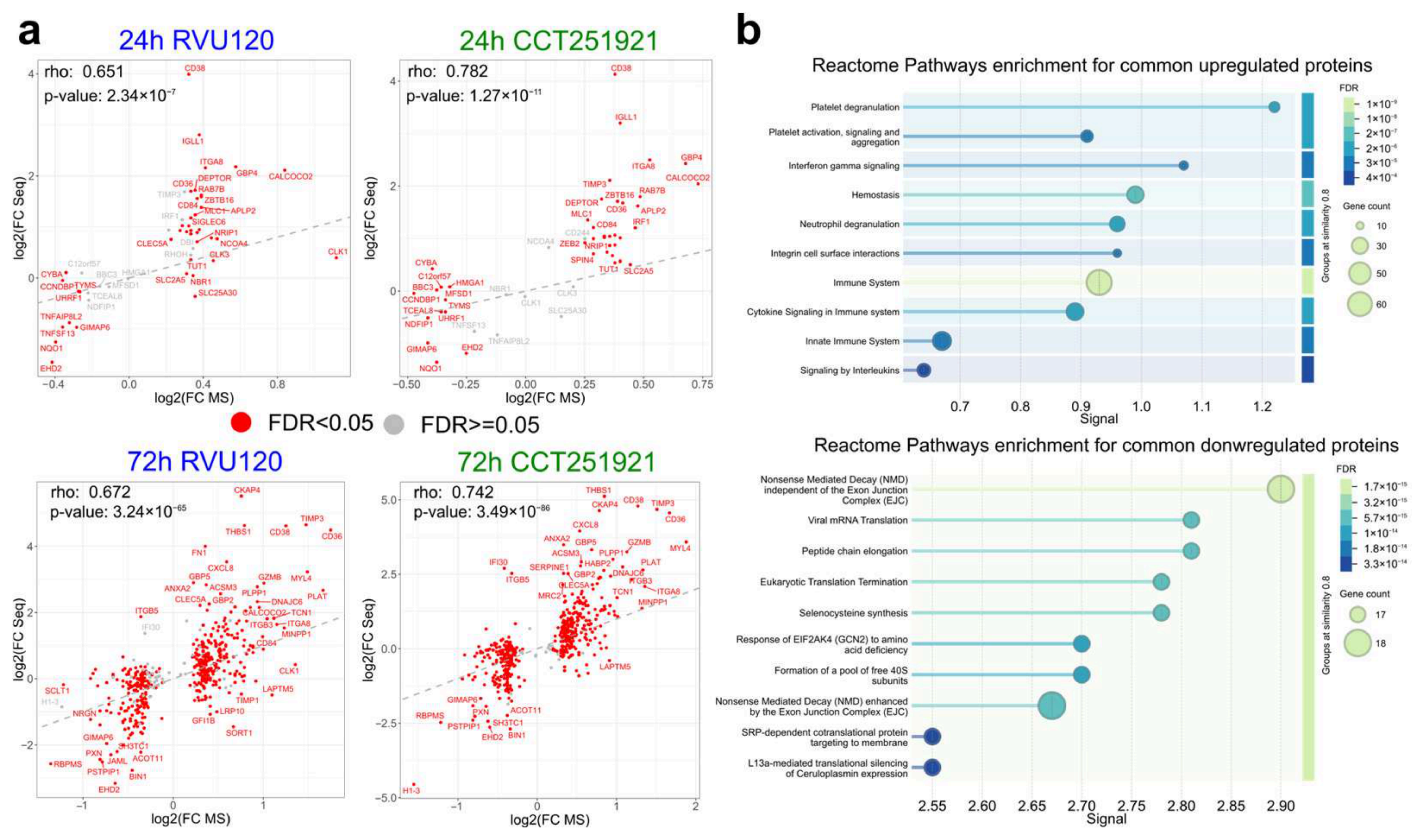
Concordant transcriptomic and proteomic responses following RVU120 or CCT251921 treatment in TEX cells. (**a**) Correlation analysis of differentially expressed proteins with their respective transcripts in the TEX cell line at 24 and 72 h after treatment with RVU120 or CCT251921 reveals significant concordance between the datasets. (**b**) Functional analysis of the commonly altered proteins in the TEX cell line at 72 h after RVU120 or CCT251921 treatment using the Reactome database implemented in the STRING tool reveals significant overrepresentation of extracellular matrix (ECM) proteins, integrins, and cell adhesion proteins.

For whole-proteome and phosphoproteome analyses, five biological replicates for each condition were prepared. The tryptic peptides were labelled with TMT reagents, and the samples were divided into portions either for a direct whole-proteome survey or phosphopeptide enrichment using Ti-IMAC beads. In the whole-proteome analysis, 6521 proteins, each with at least two peptides from MS/MS spectra (FDR ≤ 0.01) were identified. Only peptides with an unambiguous assignment to a single protein were considered (Table S3). Proteins with fully overlapping sets of peptides were “rolled up” into a single entry in the list. There were none, 40/43, and 446/481 differentially abundant proteins (FDR ≤ 0.05), respectively, at 3, 24, and 72 h post-treatment with RVU120/CCT251921 (Table S4). Of those, 32 and 428 were commonly altered at the 24 and 72 h time points, respectively. The differentiating proteins at both time points were significantly correlated with their respective transcriptomic datasets, indicating that protein-level alterations largely mirror underlying gene expression changes in the system under study (Figure 3a).

Among the set of shared differentially expressed proteins at the 72 h time point, 249 were upregulated while 178 were downregulated. Importantly, in the proteome dataset, we observed an increase in CD38, CD36 and CD84 protein expression, well-established surface markers enriched in the erythroid lineage [25,26], at 24 h and 72 h time points for both inhibitors. The functional analysis with the Reactome database of upregulated proteins, in addition to the activation of pathways observed in the transcriptome survey, revealed a significant overrepresentation of proteins belonging to extracellular matrix (ECM) proteins (FN1, COL2A1, HSPG2, THBS1), Integrins (ITGA2B, ITGA8, ITGAV, ITGB3) and cell adhesion molecules (ICAM1, ICAM2) (Figure 3b). The upregulation of these molecules is consistent with the proposed in vitro differentiation of TEX cells toward erythroid lineage, which involves recreating aspects of the erythroblastic island microenvironment to undergo proper differentiation [27]. On the other hand, the functional analysis of downregulated proteins revealed overrepresentation of proteins assigned to terms related exclusively to translational machinery and ribosome biology. The proteins driving this enrichment are predominantly large- and small-subunit ribosomal proteins. The specific downregulation of ribosomal proteins is consistent with a prior report that CDK8, via its role in Mediator-driven transcription, directly supports ribosomal gene expression programs in medulloblastoma, and its inhibition predictably reduces this output [28].

In sum, our integrated multi-omics analyses, in line with phenotypic observations, demonstrate that CDK8 inhibition with RVU120 or CCT251921 induces highly consistent, time-dependent changes at both the transcriptomic and proteomic levels in the TEX cell line. Initial transcriptional responses were characterised by activation of cholesterol biosynthesis pathways, followed by progressive induction of inflammatory gene programs and, ultimately, commitment to an erythroid lineage state. Proteomic changes significantly correlated with their corresponding transcriptomic datasets, confirming elevated expression of erythroid surface markers alongside upregulation of extracellular matrix components, integrins, and cell adhesion molecules, collectively consistent with in vitro erythroblastic island niche remodelling. Conversely, downregulated proteins were enriched for ribosomal and translational machinery components. Together, these findings demonstrate that CDK8/19 inhibition drives a coordinated, temporally structured shift from an LSC-like transcriptional state toward erythroid differentiation, providing a mechanistic framework for the phenotypic effects observed upon prolonged inhibitor treatment.

### 3.3. Nucleic Acid-Binding Proteins Are Enriched Within the Phosphoproteome Following CDK8 Inhibition in the TEX Cell Line

**Figure 4:**
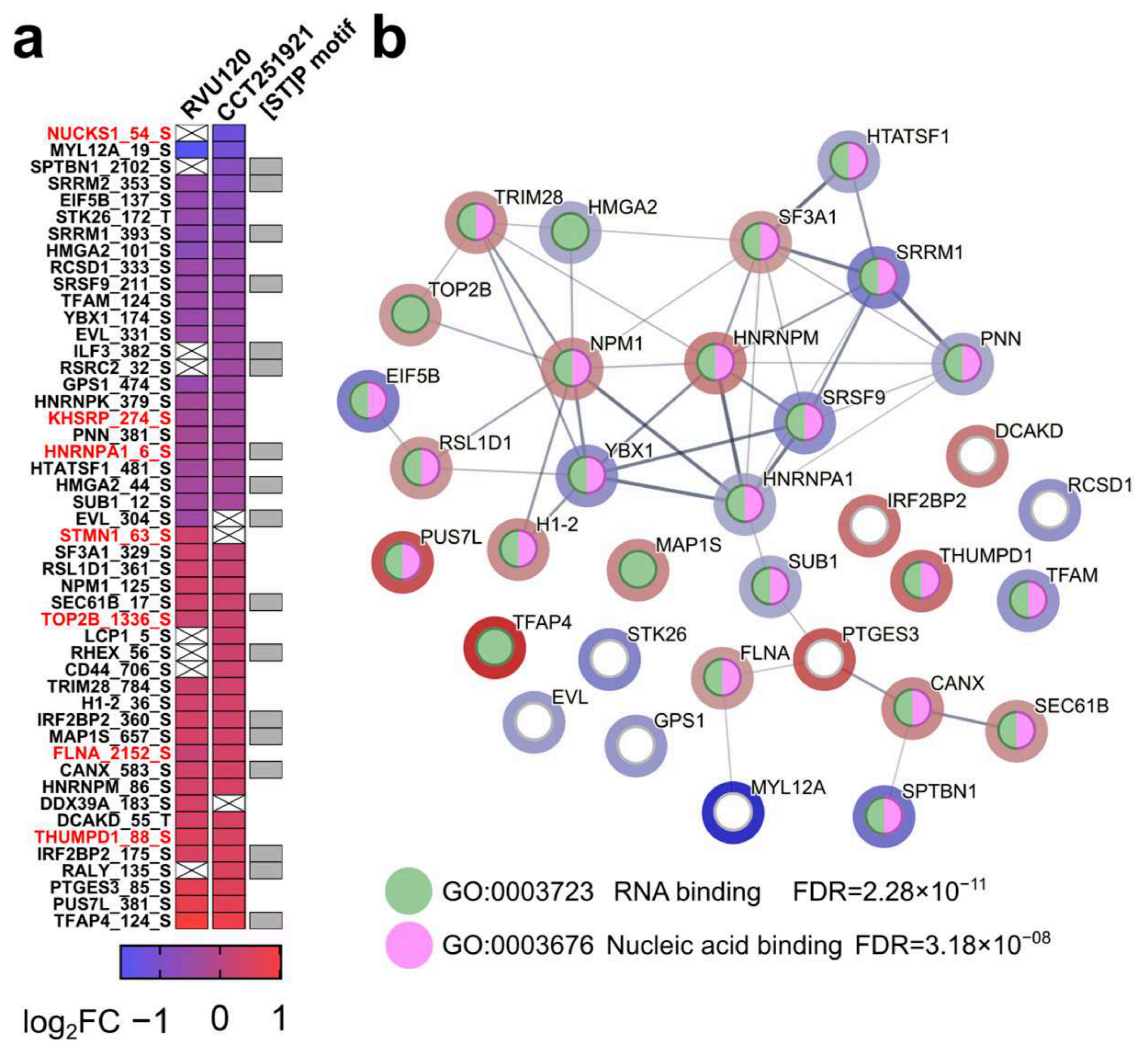
Nucleic acid-binding proteins are enriched in the phosphoproteome following CDK8 Inhibition in the TEX cell line. (**a**) Heatmap of significantly altered 33 phosphosites with concordant changes in abundance after 72 h treatment with CDK8 inhibitors. In red, the phosphosites are those shared with the work by Chen et al. [9]. (**b**) String analysis of network nodes of proteins with identified phosphosites, line thickness indicates the strength of data support. The halo colour indicates the phosphosite abundance after 72 h of CDK8 inhibition.

The impact of both CDK8 inhibitors on the TEX cell line phosphoproteome was assessed using the same samples as for the proteomic analysis, which were subsequently subjected to phosphopeptide enrichment, enabling direct normalisation of the direct site-specific quantitative values to the corresponding protein abundances from the global analysis. MaxQuant assigned sequences to 15,432 spectra, representing 6680 peptides from 1120 proteins (FDR ≤ 0.01); proteins with at least two peptides are considered reliably identified, and proteins with shared peptide lists were “rolled up” into a single entry in the list (Table S5). To narrow down the phosphosites to the most reliable ones, we applied stringent criteria. First, at least one peptide containing the specific site had to be confidently identified with an FDR ≤ 0.01, carry only one Phospho (STY) modification and exhibit correct quantitative values in each of the three TMT labelling replicates. Next, we required MaxQuant’s probabilistic modification site localisation score to be at least 0.90. Finally, when a peptide contained more than one possible modification site, the probability measure for the selected site had to be the highest among all potential sites to ensure accurate quantitative evaluation. These steps yielded 395 modification sites in 297 proteins, which were submitted for quantitative analysis (Table S6). There were none, 1/4, and 38/45 differentially abundant phosphopeptides (FDR ≤ 0.05), respectively, at 3, 24, and 72 h post-treatment with RVU120/CCT251921 (Table S7). One and 35 phosphosites were shared between the inhibitors, with concordant changes in abundance, and one and 11 exhibited CDK8 [S/T]-P-enriched motifs [8] at the 24 and 72 h time points, respectively (Figure 4a).

To investigate functional relationships among proteins whose phosphorylation is altered by CDK8 inhibition, we analysed the shared set of 33 proteins at 72 h using the STRING database. We found that this protein set forms a significantly interconnected network (PPI enrichment *p*-value: 1.17e-07), with an overrepresentation of nucleic acid-binding proteins (Figure 4b). To relate the results of our phosphoproteomic analysis following CDK8 inhibition to other studies, we compared affected phosphopeptides with recent work by Chen et al. [9], who used the HEK293 cell line with CDK8/19 kinase domain mutations or Senexin B, a CDK8 inhibitor. We identified seven commonly affected phosphoepitopes concordant with the Chen et al. study, of which three (NUCKS1_S54, KHSRP_S274 and HNRNPA1_S6) were downregulated following 72 h CDK8 inhibitor treatment in both studies, which suggests that these phosphoepitopes are CDK8 kinase phosphorylation substrates. Overall, phosphoproteomic profiling of TEX cells treated with CDK8 inhibitors identified 33 high-confidence phosphosites across 35 proteins, forming a network enriched for nucleic acid-binding proteins and including three phosphoepitopes that were concordantly downregulated with previously reported CDK8 substrates.

### 3.4. Proteomic Identification and Characterisation of the CDK8 Interactome in AML Models Designate Targets for CDK8 Inhibitor Combinatorial Therapy

**Figure 5:**
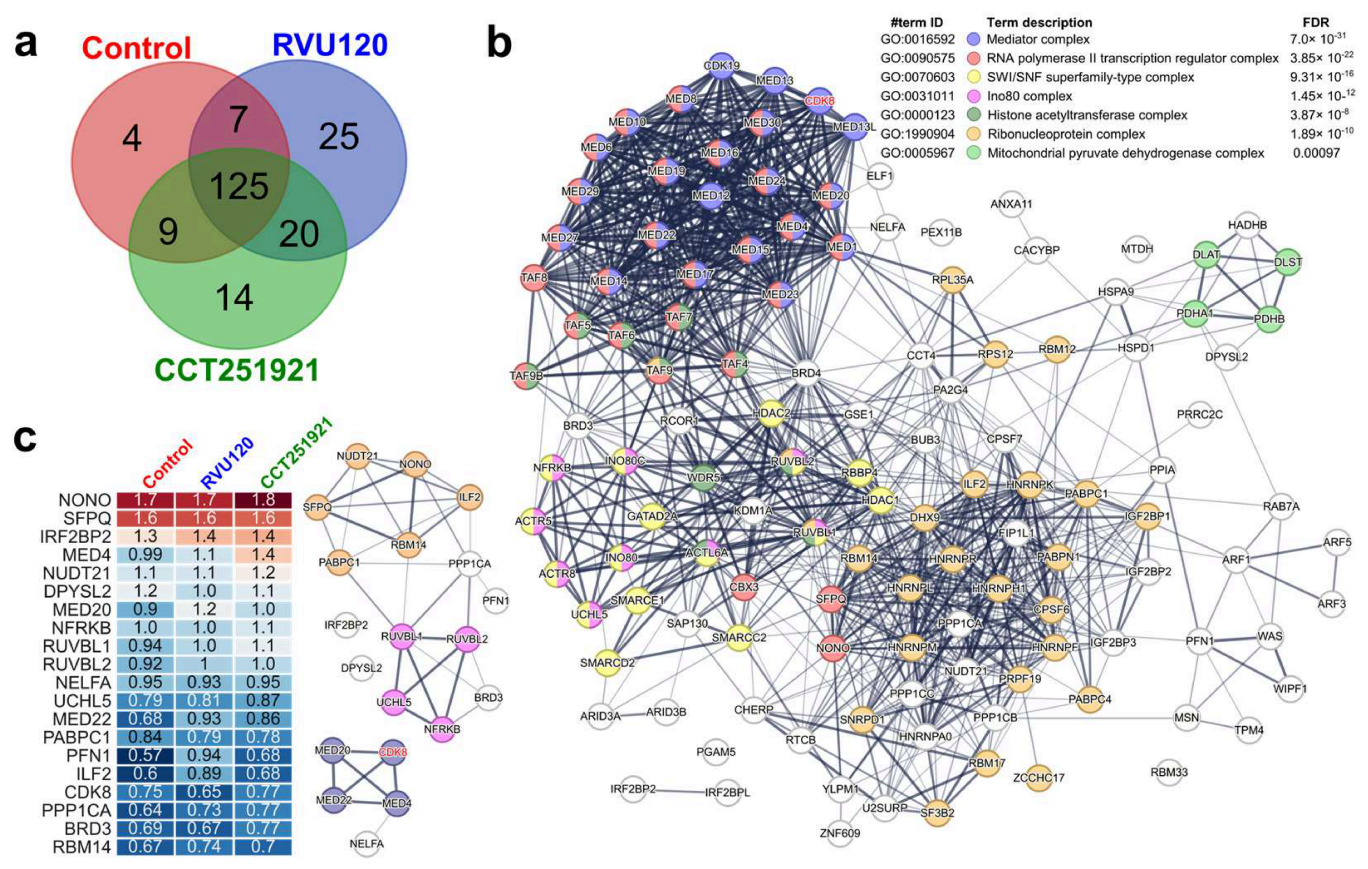
Proteomic characterisation of the CDK8 interactome in the TEX cell line. (**a**) Venn diagrams with numbers of identified CDK8 interacting proteins at control and 3 h following the RVU120 and CCT251921 treatment in the TEX cell line. (**b**) String and functional analysis of 125 core CDK8-interacting proteins; line thickness indicates the strength of data support. The functional enrichment analyses were performed within the String tool using the Reactome database and the whole genome/proteome as the statistical background. (**c**) The list of 20 most abundant CDK8-interacting proteins in the TEX cell line and their interactions based on the STRING analysis. The # abundances in the table are expressed as the APEX index.

To identify CDK8 protein core interactors and determine if they change upon CDK8 inhibitor treatment, the TEX cell line was incubated with DMSO (control) or with RVU120 and CCT251921 for 3 h, and five biological replicates per group were collected. We then used co-immunoprecipitation mass spectrometry (co-IP-MS) proteins identification, followed by label-free APEX semi-quantitative analysis of CDK8 complex constituents. MaxQuant assigned sequences to 16,130 spectra, corresponding to 2593 peptides (FDR ≤ 0.01), and identified 204 proteins by at least two peptides in at least three of the five biological replicates per group; all proteins present in IgG samples were excluded (Table S8). For functional and semi-quantitative analyses, we identified 145, 177, and 168 proteins in control, RVU120-treated, and CCT251921-treated cells, respectively, with 125 core proteins common to all three conditions (Figure 5a). Overall, 4, 25 and 14 proteins were exclusively identified in control, RVU120- and CCT251921-treated cells, respectively, while 20 proteins were common for cells treated with both CDK8 inhibitors.

To assign functional roles to the core CDK8-interacting proteins in the TEX cell line, we performed STRING analysis using the Reactome database. The CDK8 interactome was enriched for transcriptional regulatory complexes, with a predominant association with Mediator components and the RNAP2 transcription regulatory complex [11]. Beyond the transcriptional machinery, the interactome included members of the SWI/SNF chromatin-remodelling family, the INO80 complex, histone acetyltransferases (HATs), and multiple components of ribonucleoprotein complexes (Figure 5b). Direct biochemical interactions have been reported between human SWI/SNF subunits (SMARCA4 and BAF47) and the Mediator subunits MED23 and MED26 [29]. Mediator also associates with HAT complexes; for example, in mice, the Ada-Two-A-containing HAT complex interacts with Mediator to regulate non-coding RNA genes [30]. The presence of RNA-binding proteins suggests broader connections between the CDK8 network and mRNA co-transcriptional processing. Consistent with this notion, a direct interaction between CDK8 and mRNA 3′-end processing factors was recently reported in HeLa cells [31]. Notably, our co-IP-MS analysis identified the mitochondrial pyruvate dehydrogenase complex, including DLAT, PDHB, DLST, and PDHA1. These mitochondrial proteins form 2-ketoacid dehydrogenase complexes and were recently shown to be recruited to Mediator to supply a local pool of acetyl-CoA for HATs [32]. Together, these data align with previous studies of the Mediator complex interactome, indicating that overrepresented PPIs within the CDK8 interactome in TEX cells involve the RNAP2-Mediator complex, chromatin remodellers (INO80, SWI/SNF), and regulatory proteins that control transcription, RNA processing, and chromatin structure. We next focused on the most abundant proteins in the CDK8 interactome, ranking them by the semi-quantitative APEX index, to identify CDK8 partners that could be targeted with available drugs in combination therapy. The top 20 most abundant proteins identified by the STRING analysis comprised three main nodes: Mediator complex components (MED4, MED20 and MED22), the INO80 complex (NRFKB, RUVBL1/2 and UCHL5) with the associated BRD3 protein, and RNA/DNA-binding proteins (Figure 5c). The interaction of CDK8 with NFRKB and BRD3 was additionally confirmed in an independent co-IP reaction followed by Western blot (Figure S3). To assess the relevance of CDK8 protein interactors in other AML models, we surveyed these interactors using co-IP-MS in three AML cell lines (MOLM13, MOLM16, and MV4-11) and two lines derived from AML PDX models (AMLX22 and AMLX73) (Table S9). The analysis identified 39 CDK8-interacting proteins across the tested models, of which 11, including IRF2BP2, RUVBL1/2, SFPQ, NUDT21, NELFA, NONO, NFRKB, PABPC1, BRD3 and DPYSL20, were shared with the top 20 core CDK8 proteins identified in the TEX cell line. For further functional experiments, we designated the INO80 complex and BRD3, as these constituents already have available small-molecule inhibitors. Specifically, CB6644 is a selective, allosteric inhibitor of the INO80 complex RUVBL1/2 ATPases [33], whereas Pelabresib is a small-molecule BET inhibitor for the treatment of myelofibrosis [34]. In sum, the proteomic characterisation of the CDK8 interactome in several AML models defined a conserved core network of transcriptional and chromatin-remodelling factors, notably the INO80 complex and BRD3, thereby identifying these interactors as chromatin CDK8-associated partners and potential actionable targets for combinational AML therapy with CDK8 inhibitors, which was tested next.

### 3.5. Genomic Occupancy Profiling Reveals Distinct Chromatin Remodelling Programs Following CDK8 Inhibition

**Figure 6:**
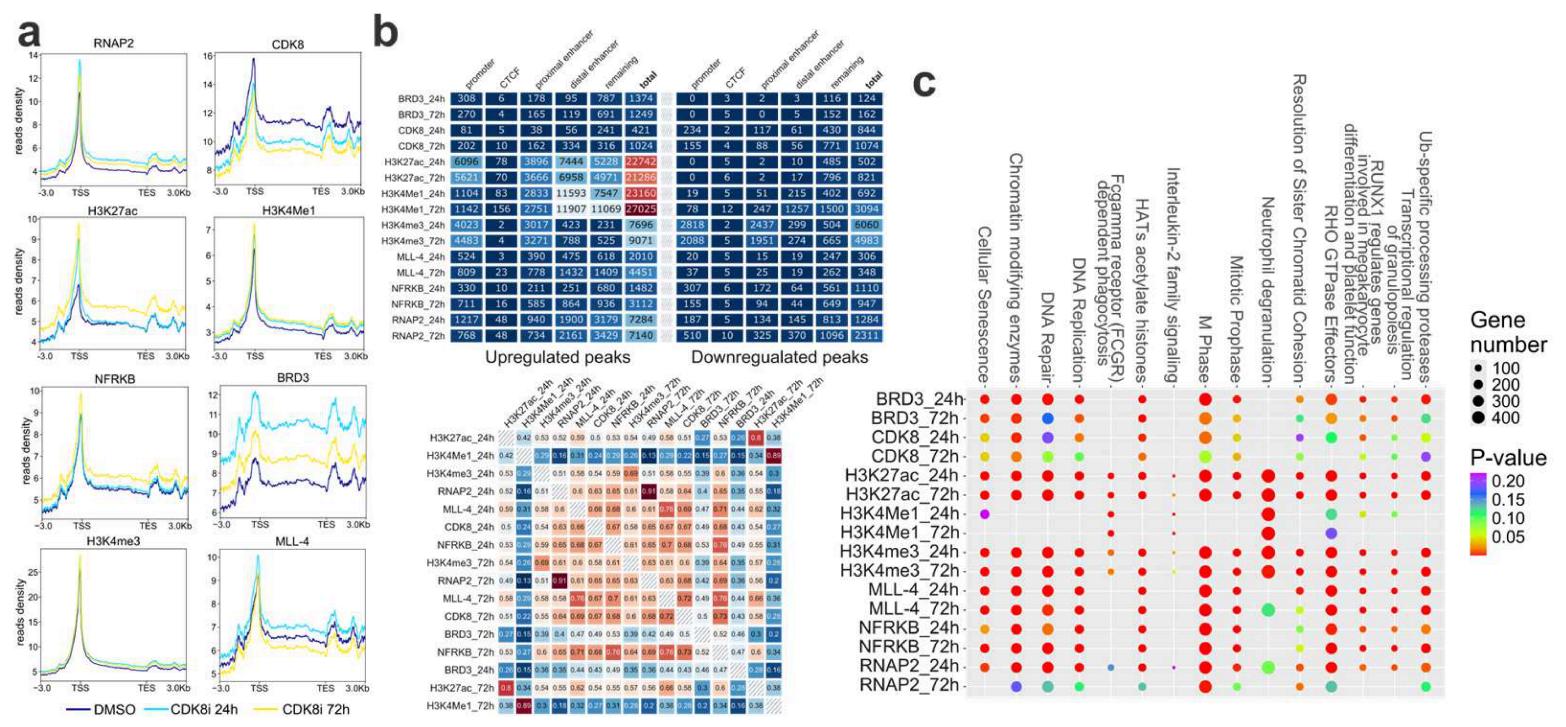
Genomic occupancy profiling reveals distinct chromatin remodelling programs following CDK8 inhibition. (**a**) Genome-wide distribution of CDK8, NFRKB, BRD3, RNAP2, MML4, and H3K27ac, H3K4me1, and H3K4me3 histone marks around transcription start sites (TSSs) and transcription end (TESs) in TEX cells following 24 h and 72 h CDK8 inhibition, illustrating loss of CDK8 binding and coordinated gain of signals for other factors after CDK8 inhibition. (**b**) Upper panel. Number of up- and downregulated MASC2 defined peaks (q-value ≤ 0.01) for each factor (BRD3, CDK8, H3K27ac, H3K4me1, H3K4me3, MLL-4, NFRKB, RNAP2) at 24 h and 72 h following CDK8 inhibition, stratified by genomic annotation (promoter, CTCF, proximal enhancer, distal enhancer). Lower panel. Pairwise Pearson correlation matrix of genomic occupancies for CDK8, RNAP2, NFRKB, BRD3, MLL4 and histone marks (H3K4me1, H3K4me3, H3K27ac) at 24 h and 72 h after CDK8 inhibition, revealing a tightly correlated MLL4–RNAP2–CDK8–NFRKB network and a distinct H3K4me1 enhancer program with weaker coupling to the transcriptional machinery. (**c**) Dot plot of Reactome pathway enrichments for differentially bound regions across CDK8, RNAP2, NFRKB, BRD3, MLL4 and histone marks (H3K4me1, H3K4me3, H3K27ac) in CDK8 inhibitor-treated TEX cells at 24 h and 72 h versus control. Dot size represents gene count (100–400 genes/pathway), while colour indicates −log10(*p*-value), with red for highly significant enrichments and blue for less significant ones.

To investigate the genomic occupancy of NFRKB and BRD3, both implicated in chromatin-mediated transcriptional control, we performed CUT&Tag assays in TEX cells. In parallel, we profiled CDK8, RNAP2, KMT2D/MLL4, and three histone modifications: H3K27ac and H3K4me1 (active enhancer markers), and H3K4me3 (an active promoter marker) at 24 and 72 h following treatment with CDK8 inhibitors. Overall, we processed eight factors across two time points and two CDK8 inhibitors in three biological replicates, yielding 108 CUT&Tag libraries. Because the transcriptomic responses to RVU120 and CCT251921 were highly similar, we merged their datasets for each protein or histone mark in pairwise comparisons with untreated cells (DMSO). The inspection of investigated factors binding around transcription start sites (TSSs) and transcription end sites (TESs) genome-wide illustrated a loss of CDK8 binding and a coordinated gain in signals for other factors following CDK8 inhibition (Figure 6a).

To functionally interpret genomic context for the observed binding changes, the occupancy peaks for each factor exhibiting differential abundance following CDK8 treatment (Table S10) were systematically annotated to established Cis-Regulatory Elements (cCREs) by the ENCODE Project Consortium [35], including promoters, CTCF-binding sites, and both proximal and distal enhancer regions. Active enhancer marks (H3K27ac, H3K4me1) showed predominantly unidirectional gains with minimal losses (Figure 6b). H3K27ac increased at ~6000 promoter-proximal peaks and ~4000 proximal plus ~7000 distal enhancer peaks at both 24 h and 72 h, indicating widespread de novo acetylation and consistent enhancer activation. H3K4me1, a mark of primed and active enhancers, accumulated at ~2800 proximal and ~11,500–12,000 distal sites with negligible loss. Together, these data indicate that CDK8 normally restrains a large repertoire of enhancers, and its inhibition releases a broad acetylation wave, consistent with CDK8’s established role within the Mediator complex as a kinase that can suppress transcriptional activation by phosphorylating Mediator subunits and limiting co-activator recruitment [11]. The active promoter mark H3K4me3 showed a more balanced, bidirectional response (4023 gained/2818 lost at 24 h; 4483/2088 at 72 h). This pattern reflects simultaneous activation of new promoters, consistent with induction of erythroid and inflammatory gene programs, and silencing of previously active sites, likely corresponding to suppression of proliferative LSC-associated genes. The net bias toward gain (approximately 1.4:1 at 24 h, 2.1:1 at 72 h) indicates that the cumulative transcriptional effect is activating, but the concurrent losses are functionally significant and represent selective repression of a distinct gene set. Transcription factor and co-regulator occupancy changes are similarly bidirectional but interpretable in the context of Mediator redistribution. CDK8 itself showed a net loss at 24 h (234 down vs. 81 up), consistent with inhibitor-mediated dissociation from active transcription sites, followed by partial redistribution by 72 h (202 up vs. 155 down), suggesting adaptive reoccupancy at a remodelled regulatory landscape. RNAP2 occupancy increased substantially at enhancers (940 proximal, 1900 distal at 24 h), indicating eRNA production and active enhancer engagement [36]. MLL4 and NFRKB both showed net gains at enhancers with high correlation to RNAP2 (r = 0.58–0.76), placing them within the core Mediator-associated transcriptional machinery that is redistributed upon CDK8 inhibition. BRD3 accumulated at hundreds of new promoters and enhancers (308 gained at 24 h, 270 at 72 h) with minimal losses, yet its weak correlation with H3K27ac (r = 0.26–0.30) and H3K4me1 (r = 0.15–0.20) indicates it occupies a specific acetylated-chromatin subset. CTCF-associated peaks showed modest changes, suggesting that CDK8 inhibition remodels cis-regulatory elements within existing topological frameworks [37]. In sum, CDK8 inhibition produces a context-dependent chromatin response: broad, net-activating enhancer priming (H3K27ac and H3K4me1 gains), concurrent activation and selective repression at promoters (bidirectional H3K4me3), and coordinated redistribution of Mediator-proximal factors (RNAP2, MLL4, NFRKB, BRD3). The net transcriptional outcome, pro-erythroid, anti-proliferative reprogramming, is coherent with this pattern, as CDK8’s release from Mediator appears to disinhibit a broad enhancer landscape while simultaneously collapsing the CDK8-dependent transcriptional programs that maintain LSC identity.

A systematic evaluation of the Reactome pathway enrichment across datasets, covering all factors at 24 and 72 h time points, revealed a landscape of biological processes (Figure 6c, Table S11). While no single pathway reached statistical significance across all conditions, the analysis highlights two main regulatory programs: a primary proliferative and transcriptional axis common to most factors, and a distinct immune-signalling signature associated with the H3K4me1 enhancer mark. Most factors (except H3K4me1) showed enrichment for pathways involved in M phase, RNA processing, such as splicing and pre-mRNA processing, and chromatin remodelling, including histone acetyltransferases and chromatin-modifying enzymes. This suggests a coordinated regulation of the cell cycle and transcriptional machinery. Conversely, regions marked by H3K4me1 are specifically enriched for immune-related pathways, such as neutrophil degranulation, interleukin signalling, and Fc-gamma receptor-mediated phagocytosis. These results imply that while active transcription supports proliferative functions, the enhancer landscape maintains a separate immune surveillance program. Overall, CUT&Tag occupancy profiling shows that inhibiting CDK8 in TEX cells induces coordinated chromatin remodelling, characterised by widespread increases in activation marks such as H3K27ac, H3K4me1, and H3K4me3, and the presence of co-regulatory factors such as RNAP2, MLL4, NFRKB, and BRD3 at promoters and enhancers. Correlation analysis indicates stable core transcriptional networks with high persistence over time (r = 0.67–0.91), whereas H3K4me1 marks a distinct, functionally independent distal enhancer landscape. Pathway analysis highlights two key regulatory programs: one associated with proliferation and transcription at promoters, and another linked to immune signalling at H3K4me1-marked enhancers. In summary, at the chromatin level, these findings support the idea that CDK8 acts as a molecular brake on chromatin accessibility. Its inhibition shifts the epigenetic landscape toward a more permissive, highly acetylated state organised into modules that promote coordinated differentiation programs. These CUT&Tag data measure the chromatin layer only; as shown by our phosphoproteomic and proteomic analyses, CDK8 inhibition additionally involves non-chromatin mechanisms (altered phosphorylation of RNA-processing factors and metabolic/SREBP reprogramming), so the overall response is not exclusively epigenetic.

### 3.6. Dual Inhibition of CDK8 and BET Bromodomains Synergistically Eradicates Leukaemia Cells in Some AML Models

**Figure 7:**
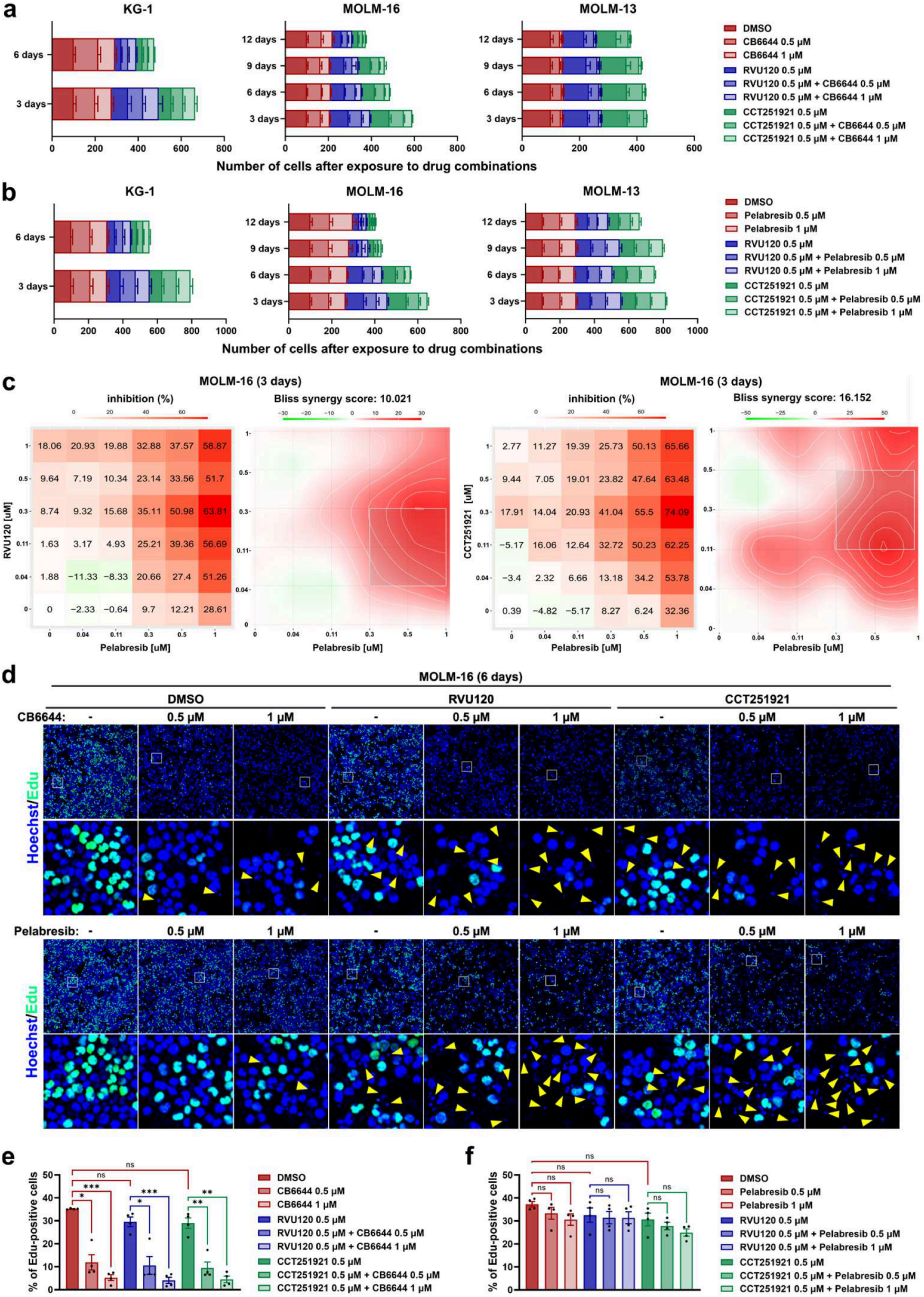
BET inhibition with Pelabresib acts synergistically with CDK8 inhibitors to potentiate their effects on MOLM-16 cell survival. (**a**,**b**) Bar graphs showing the results of high-content screening (HCS)-based analysis of KG-1, MOLM-13, and MOLM-16 cell growth upon treatment with DMSO or CDK8 inhibitors (RVU120 and CCT251921), alone or together with CB6644 (**a**) or Pelabresib (**b**). After exposure to the compounds at the indicated concentrations for the indicated days, live cell numbers were assessed using HCS microscopy of Hoechst 33342-stained cells. (**c**) Dose–response heatmap matrices of cell growth inhibition and synergy distribution visualisations showing the combined effects of treating MOLM-16 cells for 3 days with various concentrations of CDK8 inhibitors and Pelabresib. The calculated Bliss scores (+10.021 and +16.152, respectively) indicate synergy between RVU120 and Pelabresib (left) or CCT-251921 and Pelabresib (right). The analysis was performed using the SynergyFinder tool, based on HCS-assessment of live cell numbers. (**d**) Representative confocal microscopy images from the EdU incorporation assay in MOLM-16 cells treated for 6 days with DMSO, CDK8 inhibitors, Pelabresib or indicated drug combinations. Images were obtained by HCS confocal microscopy of cells stained with Hoechst 33342 dye (detecting DNA; blue), and with click-chemistry reagents (detecting Edu incorporated into replicated DNA; green). (**e**,**f**) Bar graphs showing the results of high-content screening (HCS)-based analysis of the percentage of Edu-positive cells (those that replicated their DNA) among live MOLM-16 cells treated with DMSO or CDK8 inhibitors (RVU120 and CCT251921), alone or together with CB6644 (**e**) or Pelabresib (**f**). Mean values (n = 4 ± SEM) compared statistically as indicated on the graphs. *p*-values are highlighted as: ns (*p* > 0.05), * (*p* < 0.05), ** (*p* < 0.01) or *** (*p* < 0.0001).

We reasoned that the widespread enrichment of NFRKB or BRD3 proteins at new promoters and enhancers upon CDK8 inhibition indicates a chromatin remodelling-driven formation of new properties in AML cells that survive the inhibitor treatment. Based on this, the cells that survive CDK8 inhibitor treatment could become vulnerable to interference with chromatin remodelling complexes. To precisely evaluate whether inhibiting INO80 or BRD3-related processes might offer an advantage when combined with CDK8 inhibitors, we employed a high-content screening (HCS) approach on an Opera HCS confocal microscope. This allowed us to measure the effects of single or combined treatments (for 3, 6, 9, or 12 days) on the growth and survival of three AML cell lines, KG-1, MOLM-16, and MOLM-13, which, as previously reported, vary in their sensitivity to CDK8 inhibition [6]. Consistent with our previous findings, using the HCS-based approach, we confirmed that among these cell lines, KG-1 is the most sensitive, MOLM-13 is the least sensitive, while MOLM-16 shows moderate sensitivity to RVU120 or CCT251921 treatment (Figure 7a,b).

Treatment with CB6644 alone, although it did not affect the growth of KG-1 cells, impaired the growth of MOLM-16 and MOLM-13 cells in a dose-dependent manner, with the strongest effect observed in MOLM-13 cells (Figure 7a). Conversely, Pelabresib treatment alone did not significantly impair the growth of any of the three cell lines (Figure 7b). In KG-1 or MOLM-13 cells, co-treatment with CB6644 or Pelabresib did not influence the responses to RVU120 or CCT251921 treatment (Figure 7a,b). However, we found that combining each CDK8 inhibitor with CB6644 or Pelabresib led to a notable impairment of MOLM-16 cell growth, observed after 6, 9, and 12 days of treatment with CB6644 (Figure 7a) and at all time points, starting from 3 days, with Pelabresib (Figure 7b). The growth inhibition rates of MOLM-16 cells upon single and combined treatments with CB6644 and CDK8 inhibitors indicated additive effects. Pelabresib, however, appeared to act synergistically with CDK8 inhibitors, as it did not impair cell growth alone but greatly enhanced the effects of RVU120 or CCT251921 (Figure 7b).

To verify a potential synergy between CDK8 inhibitors and Pelabresib in affecting MOLM-16 cell growth, we treated these cells for three days with various concentrations of each drug, assessed cell growth using an HCS-based approach, and performed a synergy matrix analysis, in which synergistic effects are characterised by a Bliss score > 10. This analysis confirmed that in MOLM-16 cells, Pelabresib acts synergistically with both RVU120 (Bliss Score = 10.021) and CCT251921 (Bliss Score = 16.152; Figure 7c).

**Figure 8:**
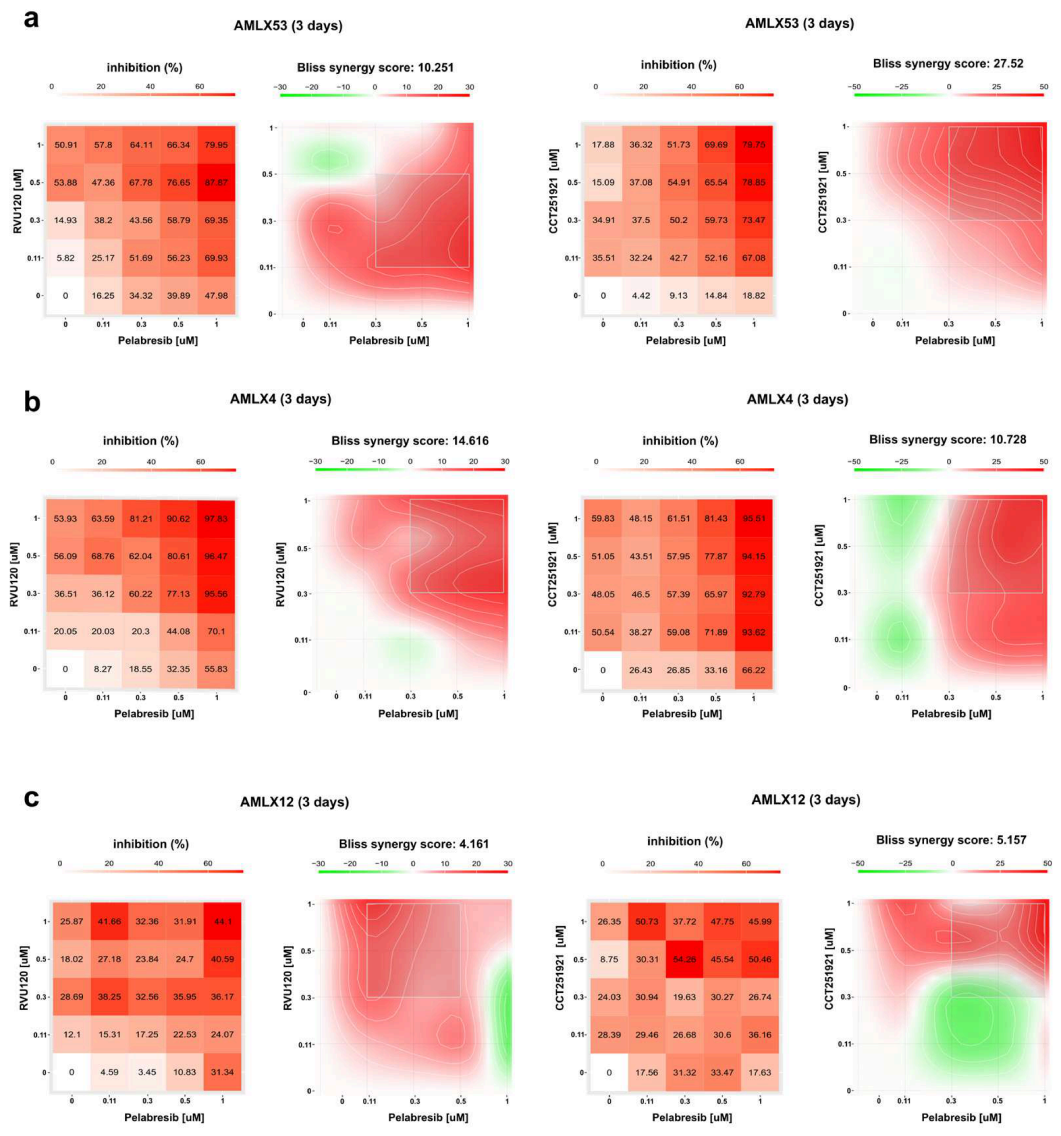
Synergy between BET and CDK8 inhibition can be observed in PDX AML models. (**a**–**c**) Dose–response heatmap matrices of cell growth inhibition and synergy distribution visualisations, showing the combined effects of treating cells from three PDX AML models. The cells were treated with various concentrations of CDK8 inhibitors and Pelabresib for 3 days. The analysis was performed using the SynergyFinder tool, based on HCS-assessed live cell counts. The results indicate synergy between RVU120 (left) or CCT251921 (right) and Pelabresib in AMLX53 and AMLX4 (Bliss scores > +10; shown in (**a**,**b**)) and additive effects between the two CDK8 inhibitors and Pelabresib in AMLX12 (Bliss scores > 0 and <+10, shown in (**c**)).

To investigate in more detail the interactions between CDK8 inhibitors and CB6644 or Pelabresib in regulating cell growth and survival, we employed HCS microscopy to assess the effects of single or combined treatments over six days on DNA content (measured by Hoechst dye staining) and DNA replication (detected by incorporated EdU) in MOLM-16 cells. We observed that RVU120 and CCT251921, although having little impact on the number of cells undergoing DNA replication, caused the appearance of numerous debris-containing dead-cell DNA (Figure 7d–f). This finding was consistent with previous results indicating that, in TEX cells, RVU120 has no effect on the cell cycle but induces apoptosis (as shown in Figure 1d). Conversely, CB6644 mainly inhibited DNA replication without causing significant cell death (Figure 7d,e). However, the combination of CB6644 with CDK8 inhibitors resulted in both outcomes—reduced DNA replication, similar to single CB6644 treatment, and increased cell death, slightly more extensive than with single CDK8i treatment (Figure 7d,e). Therefore, CDK8 inhibitors and CB6644 exert distinct effects on cell growth and survival, which may explain the additive effects observed with combined treatment. Compared to CB6644, Pelabresib had only a minor impact on DNA replication, which was not significantly enhanced by combination with CDK8 inhibitors (Figure 7d,f). However, at higher concentrations, Pelabresib led to the appearance of some dead-cell remnants, and when combined with RVU120 or CCT251921, it markedly amplified their effects on cell death (Figure 7d), indicating synergy in killing MOLM-16 cells. To validate the translational relevance of the observed synergy between CDK8 inhibitors and Pelabresib, we extended the HCS-based combination matrix analysis to patient-derived AML cells. Accordingly, we tested three PDX lines, AMLX4, AMLX12, and AMLX53. In AMLX4 and AMLX53, we identified synergy between RVU120/CCT251921 and Pelabresib (Bliss Scores > +10; Figure 8a,b), whereas in AMLX12, effects were additive (Bliss Scores < +10; Figure 8c).

Collectively, we discovered that combined CDK8 and BET inhibition represents a promising therapeutic strategy in a specific, clinically relevant subset of AML cases, in which BET inhibition potentiates CDK8 inhibitor-induced cell death.

## 4. Discussion

This study presents a multidimensional molecular portrait of CDK8 inhibition in AML stem-like cells, integrating time-resolved transcriptomics, proteomics, phosphoproteomics, interactome mapping, and chromatin profiling to establish CDK8 as a key hub in maintaining LSC-associated transcriptional programs in LSC-enriched models. The two structurally distinct inhibitors, RVU120 and CCT251921, produced highly concordant responses across all molecular layers, providing strong confidence that the observed effects are on-target. CDK8 inhibition rapidly suppressed STAT5 Ser726 phosphorylation, drove loss of the CD34^+^/CD38^−^ LSC-enriched immunophenotype, and drove cells along an erythromegakaryocytic differentiation pathway. Systematic interactome mapping identified BRD3 and the INO80 complex as conserved CDK8 partners across five AML models, while CUT&Tag profiling uncovered a chromatin remodelling program with distinct proliferative and immune-regulatory modules. Critically, these mechanistic observations translated into a pharmacologically actionable finding: CDK8 and BET inhibition synergised to eliminate AML cells in a context-dependent but reproducible manner, as confirmed in PDX models.

### 4.1. STAT5 Suppression and Erythromegakaryocytic Commitment

The time course of STAT5 Ser726/731 dephosphorylation, detectable within 6 h yet preceding phenotypic differentiation by days, establishes this event as an early and reliable pharmacodynamic marker of CDK8 target engagement, consistent with prior characterisation of RVU120 in CD34^+^ AML cell lines [6]. Importantly, cell loss in TEX cells was due to apoptosis rather than cell cycle arrest, as observed in other LSC models treated with CDK8 inhibitors. The progressive replacement of the CD34^+^/CD38^−^ LSC-enriched immunophenotype with CD38^+^, CD71^+^, and CD41^+^ surface markers over 14 days mirrors a stepwise transition through the bipotent megakaryocytic-erythroid progenitor hierarchy, rather than indiscriminate lineage bias. Because these are immunophenotypic and differentiation readouts, they indicate loss of an LSC-enriched state but, by themselves, do not establish functional eradication of LSCs, which would require serial transplantation assays [38]. This pattern mirrors findings with MK256 [39], a structurally unrelated CDK8 inhibitor that similarly induced maturation in CD34^+^/CD38^−^ LSCs models, reinforcing the view that erythromegakaryocytic commitment upon CDK8 inhibition is a reproducible, mechanism-driven outcome. The upregulation of GATA1, a master erythroid transcription factor, is mechanistically coherent in this context; prior work established that STAT5-induced erythroid differentiation in haematopoietic progenitors is directly dependent on GATA1 activity [40], placing GATA1 as a downstream effector linking STAT5 suppression to lineage commitment. Whether CDK8 suppresses GATA1 directly, through phosphorylation of GATA1-associated co-activators, or indirectly through STAT5 regulation is an important mechanistic question that targeted genetic rescue experiments can address.

### 4.2. Temporal Multi-Omics Coherence

The phased molecular response to CDK8 inhibition constitutes one of the more illuminating aspects of this work. The early activation of cholesterol biosynthesis at 3 h likely reflects metabolic reprogramming that primes cells for differentiation. Interestingly, previous work in Drosophila and mammalian models revealed that CDK8 restrains SREBP-driven lipid and cholesterol synthesis by phosphorylating SREBP, promoting its ubiquitination and degradation, thereby lowering lipogenic and cholesterogenic gene expression [41,42]. This CDK8-dependent phenomenon is therefore evolutionarily conserved, making further exploration in AML and other cancers particularly warranted, especially given the broad clinical usage of statins, well-established inhibitors of cholesterol biosynthesis. The subsequent inflammatory gene activation at 24 h is broadly consistent with the established role of inflammatory cytokine signalling in directing haematopoietic differentiation [43,44], and positions CDK8 as a suppressor of inflammatory programs in LSCs. At 72 h, the proteome extended beyond matching transcriptional changes to reveal upregulation of extracellular matrix proteins (fibronectin, collagens), integrins (ITGA2B, ITGB3), and cell adhesion molecules, features that collectively reconstitute aspects of the erythroblastic island microenvironment, which is essential for terminal differentiation [45,46]. The strong concordance between proteomic and transcriptomic datasets at both 24 and 72 h indicates that CDK8 inhibition propagates reliably from transcriptional rewiring to stable protein-level changes, a degree of coherence not always observed with kinase inhibitors and one that is important for biomarker development.

### 4.3. Phosphoproteomics: Substrates vs. Secondary Effects

The phosphoproteomic analysis identified 33 high-confidence altered phosphosites at 72 h, with no detectable changes at 3 h, a temporal profile suggesting a largely transcription-dependent, rather than direct kinase-driven, mechanism for most sites. Nonetheless, the 11 phosphosites carrying CDK8 consensus [S/T]-P motifs, particularly the three (NUCKS1_S54, KHSRP_S274, HNRNPA1_S6) that are concordant with the Chen et al. study in HEK293 cells [9], provide strong candidate direct substrates. HNRNPA1 links CDK8 activity to alternative splicing and mRNA processing [47], while NUCKS1 is a chromatin-associated regulator enriched at active promoters [48], both connect CDK8 to post-transcriptional gene regulation alongside its canonical transcriptional functions. The enrichment of nucleic acid-binding proteins across the affected phosphoproteome broadly aligns with the recently described direct interaction between CDK8 and mRNA 3′-end processing factors [31], extending this functional axis into the LSC context. The smaller phosphosite yield relative to the cortistatin A-based study [8] most likely reflects the stringent ≥ 0.90 site-localisation and quantification criteria applied here, which prioritise specificity of site assignment at the expense of sensitivity and therefore substantially reduce the number of quantifiable phosphosites. The CDK8-regulated phosphoproteome is likely broader than captured here, and identified candidates warrant orthogonal biochemical confirmation.

### 4.4. The CDK8–BRD3 Axis and Its Erythroid Dimension

The co-IP-MS data revealed a conserved CDK8 interaction network, encompassing Mediator components, the INO80 complex, and BRD3, across five AML models, nominating BRD3 as a physically proximate and functionally relevant CDK8 partner. BRD3 has been shown to bind acetylated GATA1 via its first bromodomain and co-occupy most GATA1 target loci genome-wide, with its recruitment largely independent of the histone acetylation state [49], a behaviour that directly mirrors the CUT&Tag observation that BRD3 occupancy increases at hundreds of new promoters and enhancers upon CDK8 inhibition despite only weakly correlating with H3K27ac (r ≈ 0.26–0.30). Taken together, these data suggest that BRD3 redistribution upon CDK8 inhibition could be driven by GATA1-dependent chromatin targeting, positioning BRD3 as both a molecular consequence of CDK8 inhibition and an amplifier of the erythroid differentiation program, a mechanistic connection not previously described in AML.

### 4.5. Enhancer Remodelling and Chromatin State

CDK8 inhibition triggered a broad, predominantly unidirectional gain in H3K27ac (>7000 new distal peaks) and H3K4me1 marks, with minimal losses, consistent with CDK8 acting as a molecular brake on chromatin accessibility [11]. The separation of the enhancer landscape into two functionally distinct modules is a novel observation with real biological significance: a tightly coupled RNAP2–MLL4–CDK8–NFRKB–H3K27ac axis enriched for proliferative and transcriptional programs, and a decoupled distal H3K4me1 immune-regulatory program enriched for neutrophil degranulation and interleukin signalling pathways. CDK8’s partial redistribution, declining at 24 h then recovering at 72 h, suggests adaptive reoccupancy rather than sustained eviction, which may reflect homeostatic transcriptional feedback. The minimal changes at CTCF-binding sites indicate that CDK8 inhibition remodels cis-regulatory elements within pre-existing topological domains, providing reassurance that the observed epigenetic effects are focal rather than architecturally destabilising [50]. The immune-regulatory enhancer module may represent a latent inflammatory identity normally suppressed in LSCs and liberated by CDK8 inhibition [51], potentially contributing to altered immune evasion alongside differentiation induction, a dimension worth exploring in co-culture systems with immune effector cells.

### 4.6. CDK8–BET Synergy: Mechanisms and Clinical Relevance

The pharmacologic synergy between CDK8 inhibitors and Pelabresib in MOLM-16 cells (Bliss scores of 10.0 and 16.2 for RVU120 and CCT251921, respectively) and its reproduction in two of three PDX models constitutes the most clinically actionable finding of this study. The mechanistic separation between the two drug classes, CDK8 inhibitors driving apoptosis without substantially affecting DNA replication, while Pelabresib amplified cell death in sensitive contexts, points toward convergence on a cell death threshold rather than simple anti-proliferative additivity, which matters for therapeutic index. The additive, rather than synergistic, response in AMLX12 underscores the context-dependence of this interaction. Our data do not yet define why some AML models are particularly sensitive to combined CDK8 and BET inhibition; candidate baseline determinants to test prospectively include CD34^+^/pSTAT5-high status, Mediator-kinase and BET-pathway dependency, and specific AML driver mutations, which should be evaluated by molecular profiling of responsive and non-responsive patients. Together with the established RVU120–venetoclax synergy [16], these observations provide a mechanistic rationale for a biomarker-stratified early-phase clinical trial of CDK8/BET inhibition, and motivate exploration of rational combinations with backbone regimens such as venetoclax/azacitidine (VEN/AZA), including sequential scheduling (e.g., a CDK8–BET cycle followed by a VEN/AZA cycle), in future preclinical and clinical studies. Pelabresib is already clinically active in myelofibrosis as part of the MANIFEST-2 combination regimen [34], providing a practical, safety-characterised clinical platform on which a CDK8-combination study in AML could be built.

### 4.7. Limitations and Future Directions

Several limitations need to be recognised. The TEX cell line, while well-validated as an LSC surrogate, is an engineered system that may not fully capture the genetic and epigenetic heterogeneity of primary AML. Multi-omics profiling was predominantly conducted in this single model, and validation in primary patient specimens spanning different AML molecular subclasses is needed before generalising the transcriptional and chromatin findings. The phosphoproteomic analysis, despite its rigour, applied stringent site-localisation thresholds that trade sensitivity for specificity; candidate substrates should be orthogonally confirmed by targeted kinase assays. The CUT&Tag profiling is correlational by design, and causal relationships between specific chromatin events and transcriptional outputs require perturbation experiments, such as CRISPR ablation of BRD3 or NFRKB, combined with rescue transcriptomics. A further limitation is that the effect of single-agent and combined CDK8/BET inhibition on normal HSPCs was not evaluated in this study; because a central goal of AML therapy is to eliminate LSCs while sparing normal HSPCs, the therapeutic window relative to normal CD34^+^ haematopoietic cells and colony-forming progenitors should be defined in future work. Regarding future directions, genome-wide CRISPR loss-of-function screening in synergy-sensitive versus insensitive PDX models would be the most efficient approach to identifying predictive molecular determinants of CDK8-BET co-sensitivity, potentially moving beyond pSTAT5 Ser726 as a single biomarker. Targeted depletion of BRD3 combined with CUT&Tag re-profiling in TEX and primary AML cells would directly test whether BRD3 redistribution is causally required for the observed enhancer remodelling. In vivo combination studies of RVU120 and Pelabresib in NSG-SGM3 mice engrafted with defined sensitive and insensitive PDX models are needed to establish the therapeutic window and scheduling rationale that would support early-phase clinical trial design. We emphasise that several key questions remain unresolved by the present dataset and are explicitly deferred to future work: functional confirmation that the combination eliminates bona fide LSCs (via serial transplantation or limiting-dilution assays), its impact on normal HSPCs and the resulting therapeutic window, and the molecular determinants distinguishing synergistic from additive responders. Together, these in vivo, functional, and biomarker studies would close the loop between the mechanistic observations presented here and a molecularly stratified precision-medicine approach for a clinically relevant subset of AML patients.

## 5. Conclusions

This work demonstrates that CDK8 regulates LSC-like programs and shows that inhibiting it in an AML model resembling LSCs induces a coordinated differentiation programme. This process is characterised by widespread changes in epigenetic marks, gene expression, proteins, and phosphoproteins. Our omics studies reveal that these changes are driven by a divergence in the chromatin landscape into two functional areas: one that responds to transcriptional stress by maintaining the cell cycle, and another stable enhancer programme that shifts transcriptional resources towards immune signalling and differentiation. Simultaneously, mapping the proteins that interact with CDK8 uncovers a network involving Mediator, chromatin remodellers, histone acetyltransferases, and RNA-binding proteins. Among these, INO80 and BRD3 emerge as potential targets for combination therapy. The conserved CDK8–BRD3 axis, together with the context-dependent synergistic cell killing upon dual CDK8/BET inhibition in cell lines and PDX models, offers a mechanistic rationale for a biomarker-stratified combination strategy. Its clinical translation will require identification of predictive determinants of synergy and assessment of the therapeutic window relative to normal haematopoietic cells, but is facilitated by the availability of Romaciclib, a selective CDK8/CDK19 inhibitor now in early-phase clinical trials for MDS, myelofibrosis, and R/R AML.

## Data Availability

The RNA-Seq data are available in the Gene Expression Omnibus (GEO) under the accession GSE309991. The CUT&Tag data are available in the GEO under the accession GSE319092. The MS data have been deposited to the ProteomeXchange Consortium via the PRIDE partner repository with the dataset identifier PXD074404 and 10.6019/PXD074404.

## Supplementary Materials

The following supporting information can be downloaded at: https://www.mdpi.com/article/10.3390/cells15151414/s1, Supplementary Methods: detailed protocols for Section 2.2, Section 2.5 and Section 2.8, Section 2.9, Section 2.10, Section 2.11, Section 2.12, Section 2.13, Section 2.14, Section 2.15, Section 2.16, Section 2.17 and Section 2.18; Figure S1: (a) Representative scatter plots from flow cytometric detection of incorporated BrdU and DAPI staining intensity in TEX cells treated for 3 or 6 days with DMSO or RVU120 at the indicated concentrations. Gating shows cells in various cell cycle phases (S, G2/M, G1, or G0). The results of this experiment are included in the analysis shown in Figure 1c. (b) Representative scatter plots from flow cytometric cell death assessment in TEX cells stained with Annexin V and 7-AAD after treatment with DMSO or RVU120 at indicated concentrations for 7 or 14 days. Gating indicates live cells (bottom left), early apoptotic cells (top left) and late apoptotic/necrotic cells (top right). Graphical representation of the number of cells in each population shown in Figure 1d; Figure S2: (a) Levels of STAT5, p-STAT5 (S726/S731) and GATA1 proteins in TEX cells treated for indicated times with 0.5 µM RVU120 or CCT251921. Vinculin used as a loading control. (b) Long-term viability of TEX cells treated with increasing concentrations of CCT251921 for 7, 10, 14, and 18 days. Data are mean ± SD of technical triplicates from a representative experiment (n = 3). A representative graph is shown; Figure S3: Co-IPs with mock IgG and anti-CDK8 antibody, followed by immunostaining, confirm CDK8 interactions with BRD3 and NFRKB in the TEX cell line. IPs were resolved by SDS-PAGE, then electrotransferred to a PVDF membrane and visualised by immunoblot using anti-BRD and anti-NFRKB antibodies. An amount of 10 μg of total protein extract was loaded to denote the position of proteins; Table S1: Pair-wise comparison of gene expression between control (DMSO, Column B–D) and CDK8 inhibitors (RVU120/CCT251921; Column E–G) treated TEX cell line for 3 h, 24 h and 72 h. Columns A–G contain transcript counts. “ENSEMBL” database transcript identifier; *p*adj—adjusted *p*-value from negative binomial Wald test; FC—fold change; gene_biotype—transcript type according to ENSEMBL annotation. Pair-wise comparisons for a given time point and inhibitor are arranged in Excel sheets in the following format: 3/24/72 h_inhibitor name; Table S2: Over-representation among Reactome pathways of significantly differentially expressed genes (DEGs) (*p*.adjusted < 0.05) (upregulated; downregulated) between control (DMSO) and RVU120/CCT251921 treated TEX cell line at 3 h, 24 h and 72 h. ID—Reactome pathway ID; GeneRatio—ratio of the number of differentially expressed genes associated with a given pathway and all genes associated with that pathway; BgRatio—ratio of the number of genes associated with a given pathway and all assessed genes; q-value—*p*-value adjusted for multiple hypothesis testing; geneID—list of DEGs associated with a given pathway. Reactome pathways for a time point, CDK8 inhibitor, and the direction of DEGs (up/down) used are presented in Excel sheets in the following format: CDK8i_up/down_timepoint; Table S3: List of proteins identified in the whole-proteome by LC-MS/MS analysis. Proteins with at least two peptides from the MS/MS spectra (FDR ≤ 0.01) in each of the three TMT labelling batches were considered. UniProt ACC—protein identifier in the UniProt database; Table S4: List of significantly changed proteins in the whole-proteome analysis between control (DMSO) and RVU120/CCT251921 treated TEX cell line at 24 h and 72 h. UniProt ACC—protein identifier in the UniProt database; Peptides—number of assigned peptides to a given protein; fold change; FDR—false discovery rate; Table S5: List of proteins identified in the Ti-IMAC-enriched peptides fraction by LC-MS/MS analysis. Proteins with at least two peptides from the MS/MS spectra (FDR ≤ 0.01) in each of the three biological replicates (TMT-replicates) were considered. UniProt ACC—protein identifier in the UniProt database; Peptides—the number of peptides assigned to a given protein; Seq. coverage—percent of the amino acid sequence covered by the identified peptides; Table S6: List of high-confidence phosphorylation sites selected for quantitative analysis. Phosphosites were filtered using stringent criteria requiring (i) identification of at least one peptide with FDR ≤ 0.01 containing a single Phospho (STY) modification and valid quantitative values across three TMT replicates; (ii) site localisation probability ≥ 0.90; and (iii) highest localisation probability among potential modification sites within the peptide. UniProt ACC—protein identifier in the UniProt database; Position—amino acid number with P-site in the protein; AA—type of modified amino acid; P—MaxQuant probabilistic site localisation score; Samples—number of biological replicates with identified peptide; MS/MS spectra—the number of supporting MS/MS spectra; Sequence Window—the amino acid sequence window (with the modification site at the centre); Motif—presence of the CDK motif; columns named: PhosphoSitePlus database [52], the study by Sugiyama et al. [53], and the iPTMNet database [54] contain the names of the kinases assigned to a given phosphosite by these databases; Table S7: Time-dependent changes in phosphopeptide abundance following RVU120/CCT251921 treatment. Quantitative analysis used TMT isobaric labelling, and phosphopeptide amounts were normalised to protein abundances in the studied samples. UniProt ACC—protein identifier in the UniProt database; Position—amino acid number with P-site in the protein; AA—type of modified amino acid; Sequence Window—the amino acid sequence window (with the modification site at the centre); P—MaxQuant probabilistic site localisation score; Motif—presence of the CDK motif; FC—fold change; FDR—false discovery rate; columns named: PhosphoSitePlus [52] and the iPTMNet [54]. contain the names of the kinases assigned to a given phosphosite by these databases; Table S8: List of proteins, with the number of assigned peptides (column E–S) and their semi-quantitative abundances (column T–V), identified in the CDK8 interactome in TEX cells treated for 3 h with RVU120 and CCT251921 by LC-MS/MS analysis. Proteins were considered if they met the following criteria: (1) identified by at least two peptides from MS/MS spectra with FDR ≤ 0.01, and (2) detected in at least three of five biological replicates. Semi-quantitative abundances are expressed using the APEX index. UniProt ACC—protein identifier in the UniProt database; Table S9: CDK8 interactome proteins identified by LC-MS/MS across AML cell lines and PDX models. The table lists proteins and their semi-quantitative APEX index, identified in the TEX, MOLM-16, MOLM-14, and MV4-11 cell lines, as well as in the AMLX22 and AMLX73 patient-derived xenograft (PDX) models. Proteins were considered if they were identified by at least two peptides from MS/MS spectra with FDR ≤ 0.01. UniProt ACC—protein identifier in the UniProt database; Table S10: Differential peaks for a given factor identified by MACS2 between control TEX cells and cells treated with CDK8 inhibitor for 24 h or 72 h. Each sheet contains data for a given factor and time point, in the format: factor_timepoint. chr—chromosome; start—peak start coordinate; end—peak end coordinate; length—length of a peak; Abs summit—a summit for a given peak pileup—sequencing coverage; −log10(*p*-value)—*p*-value given in logarithmic scale; fold_enrichment—fold enrichment for peak region against random Poisson distribution; −log10(q-value)—q-value (adjusted *p*-value) given in logarithmic scale; Name—unique peak id; external_gene_name—gene name assigned to the peak by annotatePeak function (grch38 database); ensembl_gene_id—ensemble ID of a gene assigned to the peak; annotation—gene feature peak overlaps; geneStrandcoordinates of a gene assigned to a given peak (chromosome, start, end, length and strand) distanceToTSS—distance of a peak to the closest transcription start site (0 if a peak overlaps TSS); Table S11: Reactome pathway enrichment analysis results from differential CUT&Tag peak genes comparing a factor, e.g., H3K27ac, and timepoint, e.g., 24 h/72 h, vs. K (control). The table lists the top pathways ranked by adjusted *p*-value, with Reactome ID, description, gene ratio/count, background ratio, *p*-value, adjusted *p*-value, q-value, and associated gene IDs.

## Abbreviations

The following abbreviations are used in this manuscript:

ACN	Acetonitrile
AML	Acute Myeloid Leukaemia
APEX	Absolute Protein Expression (semi-quantitative index)
BET	Bromodomain and Extra-Terminal domain
cCRE	Candidate Cis-Regulatory Element
Co-IP-MS	Co-Immunoprecipitation Mass Spectrometry
CUT&Tag	Cleavage Under Targets and Tagmentation
DEG	Differentially Expressed Gene
DMSO	Dimethyl Sulfoxide
EPO	Erythropoietin
eRNA	Enhancer RNA
FACS	Fluorescence-Activated Cell Sorting
FBS	Foetal Bovine Serum
FC	Fold Change
FDR	False Discovery Rate
GEO	Gene Expression Omnibus
HSPCs	Haematopoietic Stem and Progenitor Cells
HAT	Histone Acetyltransferase
HCS	High-Content Screening
LSC	Leukaemic Stem Cell
MDS	Myelodysplastic Syndrome
PCA	Principal Component Analysis
PDX	Patient-Derived Xenograft
PPI	Protein–Protein Interaction
RNAP2	Polymerase II RNA
RPKM	Reads Per Kilobase per Million mapped reads
TES	Transcription End Site
Ti-IMAC	Titanium-Immobilised Metal Affinity Chromatography
TMT	Tandem Mass Tag
TSS	Transcription Start Site
