# Supplementary material for: Integrated Multi-Omics and Interactome Analysis of CDK8 Inhibition Reveals Erythroid Differentiation Programs and Therapeutic Synergy with BET Blockade in AML
Source: Cells. 2026 Aug 4;15(15):1414. doi: 10.3390/cells15151414 (PMC13465939; doi:10.3390/cells15151414)
Supplement: Supplementary file 1 [file cells-15-01414-s001.zip › Figure S1.pdf]

a

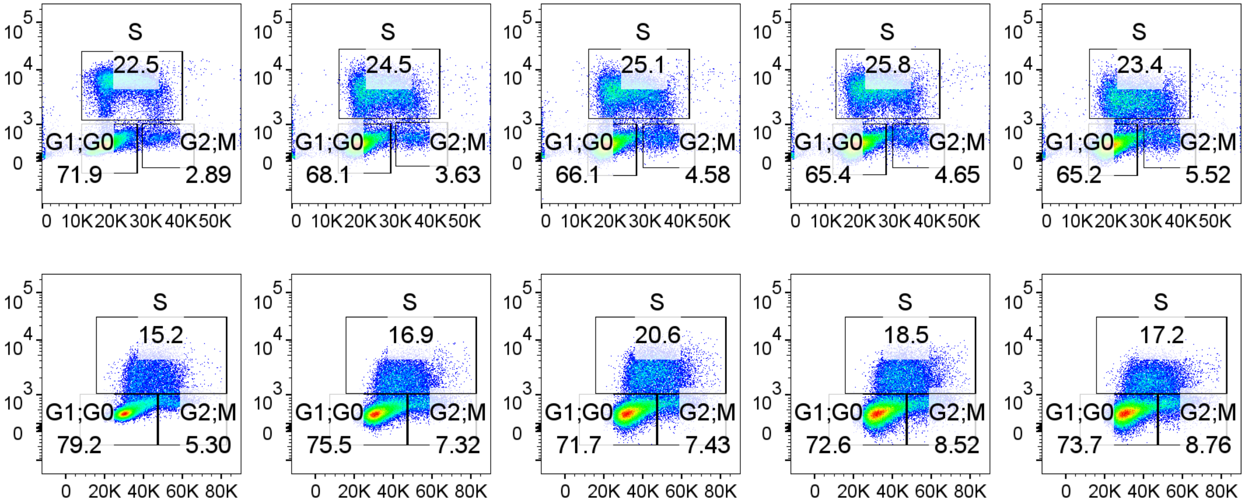
S
22.5
G1;G0
G2;M
71.9
2.89
S
24.5
G1;G0
G2;M
68.1
3.63
S
25.1
G1;G0
G2;M
66.1
4.58
S
25.8
G1;G0
G2;M
65.4
4.65
S
23.4
G1;G0
G2;M
65.2
5.52
0 10K 20K 30K 40K 50K
S
15.2
G1;G0
G2;M
79.2
5.30
S
16.9
G1;G0
G2;M
75.5
7.32
S
20.6
G1;G0
G2;M
71.7
7.43
S
18.5
G1;G0
G2;M
72.6
8.52
S
17.2
G1;G0
G2;M
73.7
8.76
0 20K 40K 60K 80K

b

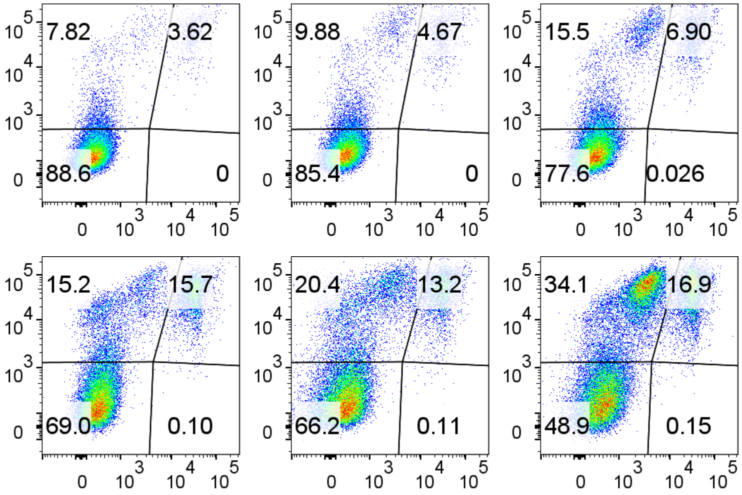
7.82
3.62
88.6
0
9.88
4.67
85.4
0
15.5
6.90
77.6
0.026
15.2
15.7
69.0
0.10
20.4
13.2
66.2
0.11
34.1
16.9
48.9
0.15
