# Supplementary material for: Integrated Multi-Omics and Interactome Analysis of CDK8 Inhibition Reveals Erythroid Differentiation Programs and Therapeutic Synergy with BET Blockade in AML
Source: Cells. 2026 Aug 4;15(15):1414. doi: 10.3390/cells15151414 (PMC13465939; doi:10.3390/cells15151414)
Supplement: Supplementary file 1 [file cells-15-01414-s001.zip › Figure S3.pdf]

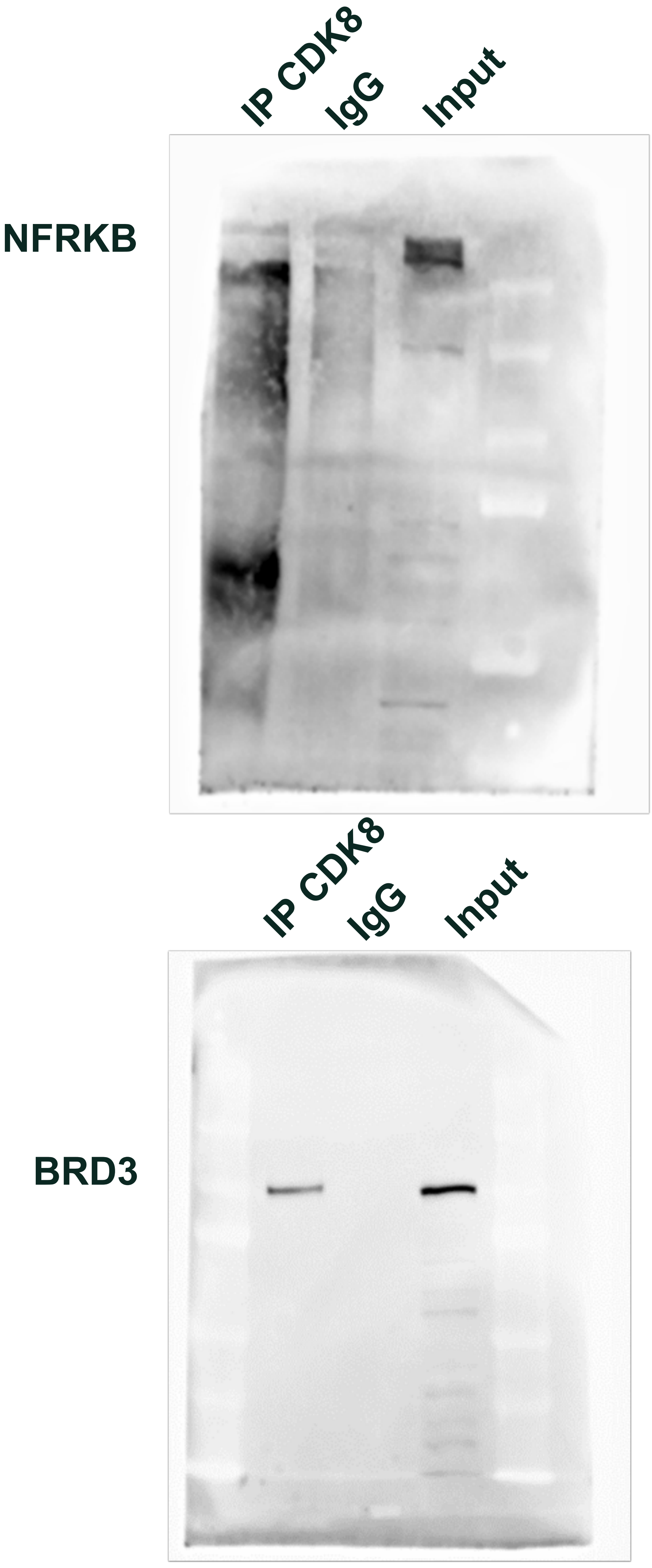
IP CDK8
IgG
Input
NFRKB
IP CDK8
IgG
Input
BRD3
