# Supplementary material for: Integrated Multi-Omics and Interactome Analysis of CDK8 Inhibition Reveals Erythroid Differentiation Programs and Therapeutic Synergy with BET Blockade in AML
Source: Cells. 2026 Aug 4;15(15):1414. doi: 10.3390/cells15151414 (PMC13465939; doi:10.3390/cells15151414)
Supplement: Supplementary file 1 [file cells-15-01414-s001.zip › Supplementary methods.pdf]

Supplementary methods

2.2 Cell Culture

All leukemia cell lines were obtained from the American Type Culture Collection (Rockville, MD, USA). TEX cells were a generous gift from Dr. John Dick and provided under the MTA with University Health Network (Toronto, Canada). Cell lines were cultured in a humidified atmosphere containing 5% CO_2 at 37°C. TEX cells were maintained in their recommended media: IMDM (Gibco, 21980-032) supplemented with 15% FBS (Gibco A5256701), 1 mM sodium pyruvate (Gibco, 11360039), 1:100 Penicillin/Streptomycin (Gibco, 15140122), 20 ng/ml SCF (R&D Systems 255-SC-050 or PeproTech 300-07), and 2 ng/ml IL-3 (R&D Systems 203-IL or PeproTech 200-03). MOLM-13, MOLM-14, MV4-11, and MOLM-16 cells were cultured in RPMI-1640 medium supplemented with 20% fetal bovine serum. KG-1 cells were cultured in IMDM medium supplemented with 10–15% FBS, 20 ng/ml SCF, and 2 ng/ml IL-3 (PeproTech, Cranbury, NJ, USA). Cells were harvested 1–2 times per week and reseeded at 4×10^5 cells/ml. All cell lines were authenticated using short tandem repeat profiling, and the absence of mycoplasma contamination was confirmed by the MycoAlert assay (Lonza LT07-518).

2.5 Flow Cytometry

TEX cells were seeded in the recommended media supplemented with EPO 2U/ml (Peprotech, 100-64) at a density of 1.2 x 10^6 cells/well in a 6-well plate and treated with 0,5 uM RVU120. Cells were re-seeded every 3-4 days and analyzed by flow cytometry at day 4, 7, 11 and 14. TEX cells were incubated with Human Fc Block (BD 564220). Following blocking, samples were stained with an anti-human antibody panel containing and isotype controls: CD71 FITC (1:20, ImmunoTools 21810713), CD34 PE (1:20, BioLegend 343506), CD38 BV510 (1:20, BioLegend 356612), CD41 AF700 (1:80, Sony Biotech 2118640), mIgG1 FITC (200 µg/µl, BioLegend 400110), mIgG1 PE (200 µg/µl, BioLegend 400122), mIgG1 BV510 (100 µg/µl, BioLegend 400172), mIgG1 AF700 (500 µg/µl, Sony Biotech 2600720). Additionally, samples were stained with LIVE/DEAD Fixable Near-IR (Invitrogen L34976) to assess cell viability. Flow cytometry analysis was performed on the BD LSR Fortessa cytometer and using FlowJo software (v.10.8.1).

2.8 Protein immunoprecipitation for Western blotting

Tex cells (5×10^6 cells per sample) were resuspended in 1 mL of ice-cold IP buffer (150 mM NaCl, 50 mM Tris-HCl (pH 7.5), 5 mM EDTA (pH 8), 1% Triton X-100, 0.5% NP40, with protease and phosphatase inhibitors (Thermo Scientific, 78441). The cells were kept on ice for 5 minutes, then sonicated in aliquots (4 × 250 µL) using a Bioruptor Pico (Diagenode) at high intensity for 10 minutes. The lysates were clarified by centrifugation at 10,000 × g for 15 minutes at 4°C. To the cell lysates, 5 µg antibody was added: NFRKB (St John's Laboratory, STJ112163-100), NFRKB recombinant (Invitrogen, MA5-55502), NFRKB (Bethyl, A301-459A); BRD3 (Santa Cruz Biotechnology, sc-81202), CDK8 (Bethyl, A302-500A), gently resuspended by pipetting, and incubated overnight at a cold room (4°C) with rotation. To the cells (Ag)-Ab was added 50 µl Dynabeads (Invitrogen, 10001D) and incubated for 4h at RT with rotation.

Afterwards, the tubes were placed on the magnet, and the supernatant (flow-through) was transferred to separate tubes. The samples (Ag-Dynabeads-Ab) were gently resuspended in 50 μl IP buffer, gently resuspended by pipetting, and 12.5 μl 4x Laemmli Buffer (Bio Rad 1610747) was added, boiled (5 min 99°C). Samples were separated on the magnet, and the supernatant was transferred to a fresh tube. The samples were stored at -20°C.

2.9 Western blotting

Cells were lysed in a RIPA buffer (Thermo Scientific 89900) supplemented with phosphatase and protease inhibitors (Thermo Scientific 78444). The lysates were clarified by centrifugation and quantified using the Bradford assay (Sigma B6916). Equal amounts of whole-cell lysates were loaded on 4-15% tris-glycine gels (Bio-Rad 5678085), then transferred to PVDF membranes. Immunoblotting was performed using antibodies: NFRKB (1:1000 Bethyl A301-459A), BRD3 (1:1000 Santa Cruz Biotechnology, sc-81202), CDK8 (1:500 Santa Cruz Biotechnology, sc-1521), STAT5 (1:1000, CST 25656), p-STAT5a/b S726/731 (1:1000, Active Motif 39597), p-STAT5 Y694 (1:1000, CST 9359), Actin (1:8000, Sigma A2066), Vinculin (1:15000, Sigma V9131). Next, the membranes were washed with PBST buffer (4×) and incubated with appropriate HRP-conjugated secondary antibodies: goat anti-rabbit (Abcam, ab97051), goat anti-mouse (Abcam, ab6789) and donkey anti-goat (Abcam, ab97110). Proteins were visualised using the ChemiDoc MP Imaging System (Bio-Rad).

2.10 Proteins digestion

Cell pellets were reconstituted in a lysis buffer containing 20% trifluoroethanol (TFE), 10 mM tris(2-carboxyethyl)phosphine (TCEP), and 100 mM triethylammonium bicarbonate (TEAB). Lysis was performed by sonication in an ultrasonic bath for 30 minutes, followed by vortexing at 1000 rpm for 1 hour, and incubation at 60°C for 1 hour. Cysteine residues were blocked by incubating the samples with 30 mM S-methylmethanethiosulfonate (MMTS) for 10 min at room temperature. Before digestion, samples were diluted with 100 mM TEAB to reduce the TFE concentration to 8%. Proteolytic digestion was performed overnight at 37°C using 2 μg of trypsin (Promega GmbH, Mannheim, Germany). Following digestion, peptides were purified using 10 mg Oasis HLB columns (Waters). The cartridges were preconditioned with 1 ml of methanol and 1 ml of 0.1% formic acid (FA) in water. After sample loading and washing with 1 ml of 0.1% FA, peptides were eluted with 200 μl of 75% acetonitrile (ACN) and subsequently dried in a SpeedVac concentrator. The dried peptides were reconstituted in 100 mM TEAB, and their concentrations were determined using the Pierce™ Quantitative Colourimetric Peptide Assay (Thermo Fisher Scientific). Equal amounts of peptides were mixed to prepare an internal standard sample (IS) used for inter-TMT normalisation. 30 μg of peptides from each sample and IS in 92 μl of 100 mM TEAB were labelled with TMTpro 18plex (Thermo Fisher Scientific) tags in 40 μl acetonitrile for 1 hour on a vortex. The reaction was quenched by the addition of 8 μL 5% hydroxylamine. The labelling efficiency was checked (99.5% for all three sets) and samples were

combined and desalted using four 30 mg Oasis HLB columns (Waters) per TMT set. Cartridges were preconditioned with methanol and 2% ACN with 0.1% FA. After the sample was loaded and rinsed with 2% ACN and 0.1% FA, peptides were eluted from the columns with 400 µl 90% ACN and 0.1% FA. Aliquots were pulled and dried in a Speedvac.

2.11 Reversed-phase peptide fractionation at high pH

A total of 540 µg of peptides for each TMT set was reconstituted in 500 µl of 10 mM AH with 2% ACN. TMT-labelled peptides were fractionated using high-pH reverse-phase chromatography on an XBridge Peptide BEH C18 column (4.6 × 250 mm, 130 Å, 5 µm; Waters). Separation was performed at a flow rate of 0.8 ml/min over 64 min using a Waters Acquity UPLC H-Class system. The mobile phases consisted of water (A), ACN (B), and 100 mM AH (C), with phase C maintained at a constant 10% throughout the gradient. Fractions were collected every minute, starting from the fourth minute of the run. The following gradient was applied: 5–8% solvent B over 3 min, 8–15% over 9 min, 15–25% over 22 min, 25–33% over 10 min, 33–50% over 8 min, 50–90% over 6 min, followed by a 4 min isocratic hold at 90% B, and final column equilibration at 3% B for 2 min. Peptide elution was monitored at 214 nm. The collected 60 fractions were dried in a SpeedVac concentrator and reconstituted in Evosep solvent A (0.1% FA in water) by vortexing for 30 min, followed by sonication for 30 min. Fractions were concatenated by pooling every 30th fraction, yielding 30 samples per TMT set for LC-MS analysis. 2 µg of peptides from each concatenated fraction was loaded onto Evotips precolumns, as described in the LC-MS analysis section. The remaining sample from each TMT set was pooled, cleaned with 30 mg Oasis HLB columns, dried in a SpeedVac concentrator and subjected to phosphopeptide enrichment.

2.12 Phosphopeptide enrichment with Ti-IMAC

Phosphopeptide enrichment was performed using MagReSyn Ti-IMAC beads (Resyn Biosciences) following the manufacturer's protocol. Briefly, 480 µg of desalted TMT-labelled peptides per set was divided into two equal parts. Each portion was dissolved in 200 µl of Loading Buffer (0.1 M glycolic acid in 80% ACN and 5% TFA) and incubated with 50 µl of magnetic beads for 20 min at room temperature with gentle vortexing. After incubation, the supernatant was discarded, and the beads were washed with Wash Buffer to remove non-specifically bound peptides. Phosphopeptides were eluted sequentially with three additions of 100 µl 1% AH elution buffer containing 0%, 10%, and 20% ACN. The pulled eluates were dried in a SpeedVac concentrator and reconstituted in 50 µl of 0.1% FA.

2.13 Protein immunoprecipitation for Mass spectrometry

2.13.1 Preparation of Dynabeads A for Single IP Reaction.

50 µL of Dynabeads A (Invitrogen, 10001D) were placed on a magnet to separate them from the solution, and the supernatant was removed. The beads were washed 3 times with PBST (PBS with 0.02% Tween 20). 5 µg of the target antibody was added to the Dynabeads A (beads with IgG for background control and beads with the target antibody; unspecific rabbit IgG – Vector Labs, I-1000; 1 µg/µL or CDK8 - #A302-500A; Bethyl;

200 μg/mL). The beads with the antibody were incubated with rotation at room temperature for 30 minutes, then placed on the magnet, and the supernatant was removed. The beads were washed once by gentle pipetting with PBST. The crosslinking reaction was prepared with a 5 mM BS3 (Pierce, 21585) solution in CB (20 mM HEPES). The antibody-coupled Dynabeads A were washed twice with 200 μL CB, placed on the magnet, and the supernatant was discarded. The antibody-coupled Dynabeads A were resuspended in 300 μL of 5 mM BS3 and incubated at room temperature for 30 minutes with tilting rotation. The crosslinking reaction was quenched by adding 15 μL of QB (1 M Tris-HCl, pH 7.5) and incubating at room temperature for 15 minutes with tilting rotation. The cross-linked antibody-coupled Dynabeads A were washed three times with 400 μL PBST.

2.13.3 Protein Immunoprecipitation from AML cell lines and PDX cell models.

Cells pellets (5×10^6 cells per sample) were resuspended in 1 mL of ice-cold IP buffer (150 mM NaCl, 50 mM Tris-HCl (pH 7.5), 5 mM EDTA (pH 8), 1% Triton X-100, 0.5% NP40, with protease and phosphatase inhibitors). The cells were kept on ice for 5 minutes, then sonicated in aliquots (4 × 250 μL) using a Bioruptor Pico (Diagenode) at high intensity for 10 minutes. The lysates were clarified by centrifugation at 10,000 × g for 15 minutes at 4°C. Antibody-coupled Dynabeads A were washed once with IP buffer, placed on a magnet, and the supernatant was removed. The cell lysates were added to the IgG-Dynabeads A complex, gently resuspended by pipetting, and incubated for 30 minutes at 4°C with rotation. Afterwards, the tubes were placed on the magnet, and the supernatant (flow-through) was transferred to the CDK8-Dynabeads A complex, which was gently resuspended by pipetting and incubated for another 30 minutes at 4°C with rotation. The samples (Dynabeads-Ab-Ag) were placed on a magnet, washed 3 times with 200 μL of 150 mM Tris-HCl, pH 7.6, and then separated on the magnet. The Dynabeads-Ab-Ag complex was gently resuspended in 50 μL of 200 mM formic acid, placed on a magnet, and the supernatant was transferred to a fresh tube. The samples were stored at -80°C.

2.14 Mass spectrometry

Co-IP samples, as well as global fractions and phospho-enriched TMT samples, were analysed using an Evosep One (Evosep Biosystems) coupled to an Orbitrap Exploris 480 mass spectrometer (Thermo Fisher Scientific). Samples were loaded onto disposable Evotips C18 trap columns according to the manufacturer's instructions. One-third of each co-IP sample, or 20 μl of each TMT sample, was loaded. Evotips containing phosphopeptides were additionally washed four times with 3% ACN. Peptides were separated on an EV1106 analytical column (Dr Maisch C18 AQ, 1.9 μm, 150 μm ID, 15 cm; Evosep Biosystems). Global TMT samples were analysed at 500 nl/min using a 44 min gradient (30 samples per day), whereas phospho-enriched TMT and co-IP samples were analysed at 220 nl/min using an 88 min preformed gradient (15 samples per day). Data were acquired in positive ion mode using data-dependent acquisition. MS1 spectra were recorded at 60000 resolution

with a normalised AGC target of 300% and automatic maximum injection time. The scan range was 300–1700 m/z for TMT samples and 300–1600 m/z for co-IP samples. For TMT samples, MS2 spectra were acquired at 30000 resolution with a standard normalised AGC target and automatic maximum injection time. The top 25 precursors were selected within a 1.2 m/z isolation window and analysed in TurboTMT mode ("TMTpro Reagents"). For co-IP samples, MS2 resolution was 15000, and the top 40 precursors were selected within a 1.6 m/z isolation window. Dynamic exclusion was set to 20 s (±10 ppm), and the precursor intensity threshold was 5e3. A precursor fit threshold of 70% was applied to TMT samples. Precursors were fragmented by HCD with a normalised collision energy of 30%. The spray voltage was 2.1 kV, the funnel RF level 40, and the heated capillary temperature 275°C.

2.15 Mass spectrometry data analysis

Offline recalibration and peptide and protein identification were performed using the MaxQuant/Andromeda software suite [1] (version 2.0.1.0) against the Homo sapiens SwissProt database (version 2022_03). The search included tryptic-generated peptides, with methylthio (C) set as a fixed modification and oxidation (M) as a variable modification. Phosphorylation (STY) was also selected as a variable modification for phosphoproteome analysis.

A reverse database was used for target/decoy-based statistical validation, with peptide and protein FDR set to 0.01. All peptides derived from common contaminant proteins were filtered from the results. Also, proteins with fewer than 2 unambiguously assigned peptides and those identified by peptides from other proteins were excluded from further analysis. Proteins matching the same set of peptides were clustered into metaproteins. MaxQuant results processing was performed in MScan, a software tool available at http://proteom.ibb.waw.pl/mscan.

Quantitative analysis of the whole-proteome and phosphoproteome of TEX cells included only proteins identified by at least two unique MS/MS-confirmed peptides in every TMT labelling repeat. Relative protein abundances were computed using reporter-ion MS2 quantification in MaxQuant and processed using the internal reference scaling (IRS) algorithm for labelling batch-effect elimination [2]. To minimise the influence of other non-biological sources of variation on the data, the log-transformed abundances were further normalised using a locally weighted regression smoother (LOESS) [3]. The results of co-immunoprecipitation experiments were analysed using a semi-quantitative spectral-counting approach and summarised using the APEX index [4].

Phosphoproteome quantitative analysis was based on unique peptides with a single phosphorylation site, identified by MS/MS and correctly quantified across all TMT labelling replicates. The threshold for MaxQuant's site-localisation probability was set to 0.90, and only the site with the highest probability was analysed for peptides with multiple potential modification localisations. The obtained site-specific quantitative values were referenced to appropriate protein abundances from the global analysis. [S/T]-P motif in the peptide sequence and kinases associated with phosphorylation sites were from the PhosphoSitePlus

database [5], the study by Sugiyama et al. [6], and the iPTMNet database [7].

A resampling-based t-statistic test was used to identify differentially expressed proteins and phosphosites in pairwise comparisons. The resulting p-values were corrected for multiple hypothesis testing using the two-step Benjamini-Hochberg procedure to control the false discovery rate (FDR) [8]. The adjusted p-values and fold-change (FC) thresholds were set to 0.05 and 1.25, respectively. Unsupervised principal components analysis (PCA) was also used to evaluate the relationships among the studied samples. Statistical data analysis utilised the MATLAB-based MStat application (available at https://proteom.ibb.waw.pl/mstat).

2.16 Cut&Tag libraries preparation and sequencings

TEX cells were incubated in the presence of CDK8 kinase inhibitors (RVU120 or CCT251921 at 0.5 μM) and harvested after 24 h and 72 h. The Cut&Tag experiment was performed on 300,000 TEX cells per antibody, following the manufacturer's recommendations (CUT&Tag-IT™ Assay Kit, Active Motif, Catalogue No. 53165 or 53171). For the experiment, we used the following antibodies: H3K27ac (Active Motif, cat. no. 91193); H3K4me1 (Active Motif, cat. no. 39635); H3K4me3 (Active Motif, cat. no. 39060); RNAP2 CTD (Abcam, cat. no. Ab5095); MLL-4 (Millipore, cat. no. ABE1867); CDK8-500 (Bethyl, cat. no. A302-500); BRD3 (Invitrogen, cat. no. PA530263); NFRKB (Bethyl, cat. no. A301-459A). Prior to sequencing, all samples were adjusted to 10nM, mixed in equal amounts, and divided into two pools: pool 1 (S13662) and pool 2 (S13663). Library sequencing was performed using the paired-end 75 bp method on the NovaSeq 6000 instrument at CeGaT.

2.17 Cut&Tag data analyses

Raw sequencing reads were subjected to quality control and adapter trimming using Trimmomatic (version 0.39) [9]; with the parameters SLIDINGWINDOW:4:20 and MINLEN:20. High-quality reads were subsequently aligned to the Homo sapiens reference genome (GRCh38) using Bowtie2 [10] under default mapping conditions. Optical duplicates were identified and removed with the MarkDuplicates tool from the GATK package (version 4.2.3.0) [11], applying default settings except for *OPTICAL_DUPLICATE_PIXEL_DISTANCE* set to 12,000. Following duplicate removal, genome-wide coverage tracks were generated using bamCoverage [12] (version 3.5.1) with RPKM normalisation to enable comparison across samples. Enrichment peaks were identified using MACS2 [13] (version 2.2.7.1) with default peak-calling parameters, based on the GRCh38 reference genome assembly. Peaks were associated with genomics features with the ChIPpeakAnno package [14] (version 3.36.1). Functional annotation of differential peaks was conducted with ReactomePA package [15] (version 1.46.0). Binding profiles were prepared using DeepTools [12] (version 3.5.1).

2.18 High-content microscopy analyses and synergy testing

To analyse cell growth, the established AML cell lines (KG-1, MOLM-13 and MOLM-16) as well as PDX-derived AML lines (AMLX4, 12 and 53)

grown in suspension were plated in 96-well culture plates in triplicates at 20,000 cells (established lines) or 40,000 cells (PDX-derived lines) per well in 160 μl of their culture medium. Cells were exposed to single or combined treatments with DMSO, CDK8 kinase inhibitors (RVU120 or CCT251921), BET inhibitor (pelabresib) or RUVBL1/2 complex inhibitor (CB6644). At the designated time points, the suspension cells were attached to μCLEAR® black 96-well plates (Greiner) precoated with Poly-D-Lysine (Gibco), then fixed with 4% formaldehyde and stained with Hoechst 33342 (10 μg/ml). For analysis of DNA replication MOLM-16 cells were treated with 10 μM EdU (Lumiprobe) for 1 hour prior to fixation. The incorporated EdU was detected by click chemistry staining with AF 488 azide (Lumiprobe) according to the manufacturer's instructions. In each case, the stained cells were imaged with the Opera Phenix (Revvity) high-content imaging system, at the IN-MOL-CELL facility of the International Institute of Molecular and Cell Biology in Warsaw (IIMCB), using a 10x objective and confocal mode. Live cell counts were assessed based on the intensity, morphology and texture properties of Hoechst 33342-stained DNA, whereas the percentages of EdU-positive cells were calculated based on AF 488 azide staining intensity. All quantitative analyses were performed using the Harmony 4.9 software (Revvity). Functional interactions, such as additive or synergistic effects, between CDK8 inhibitors and pelabresib or CB6644 were analysed using the Synergy Finder 3.0 tool based on live cell counts.

References

1. Tyanova, S.; Temu, T.; Cox, J. The MaxQuant Computational Platform for Mass Spectrometry-Based Shotgun Proteomics. *Nat. Protoc.* **2016**, *11*, 2301–2319, doi:10.1038/nprot.2016.136.

2. Plubell, D.L.; Wilmarth, P.A.; Zhao, Y.; Fenton, A.M.; Minnier, J.; Reddy, A.P.; Klimek, J.; Yang, X.; David, L.L.; Pamir, N. Extended Multiplexing of Tandem Mass Tags (TMT) Labeling Reveals Age and High Fat Diet Specific Proteome Changes in Mouse Epididymal Adipose Tissue. *Mol. Cell. Proteomics MCP* **2017**, *16*, 873–890, doi:10.1074/mcp.M116.065524.

3. Bolstad, B.M.; Irizarry, R.A.; Astrand, M.; Speed, T.P. A Comparison of Normalization Methods for High Density Oligonucleotide Array Data Based on Variance and Bias. *Bioinformatics* **2003**, *19*, 185–193, doi:10.1093/bioinformatics/19.2.185.

4. Braisted, J.C.; Kuntumalla, S.; Vogel, C.; Marcotte, E.M.; Rodrigues, A.R.; Wang, R.; Huang, S.-T.; Ferlanti, E.S.; Saeed, A.I.; Fleischmann, R.D.; et al. The APEX Quantitative Proteomics Tool: Generating Protein Quantitation Estimates from LC-MS/MS Proteomics Results. *BMC Bioinformatics* **2008**, *9*, 529, doi:10.1186/1471-2105-9-529.

5. Hornbeck, P.V.; Zhang, B.; Murray, B.; Kornhauser, J.M.; Latham, V.; Skrzypek, E. PhosphoSitePlus, 2014: Mutations, PTMs and Recalibrations. *Nucleic Acids Res.* **2015**, *43*, D512-520, doi:10.1093/nar/gku1267.

6. Sugiyama, N.; Imamura, H.; Ishihama, Y. Large-Scale Discovery of Substrates of the Human Kinome. *Sci. Rep.* **2019**, *9*, 10503, doi:10.1038/s41598-019-46385-4.

7. Huang, H.; Arighi, C.N.; Ross, K.E.; Ren, J.; Li, G.; Chen, S.-C.; Wang, Q.; Cowart, J.; Vijay-Shanker, K.; Wu, C.H. iPTMnet: An Integrated Resource for Protein Post-Translational Modification Network Discovery. *Nucleic Acids Res.* **2018**, *46*, D542–D550, doi:10.1093/nar/gkx1104.

8. Benjamini, Y.; Hochberg, Y. Controlling the False Discovery Rate: A Practical and Powerful Approach to Multiple Testing. *J. R. Stat. Soc. Ser. B Methodol.* **1995**, *57*, 289–300, doi:10.1111/j.2517-6161.1995.tb02031.x.

9. Bolger, A.M.; Lohse, M.; Usadel, B. Trimmomatic: A Flexible Trimmer for Illumina Sequence Data. *Bioinformatics* **2014**, *30*, 2114–2120, doi:10.1093/bioinformatics/btu170.

10. Langmead, B.; Salzberg, S.L. Fast Gapped-Read Alignment with Bowtie 2. *Nat. Methods* **2012**, *9*, 357–359, doi:10.1038/nmeth.1923.

11. McKenna, A.; Hanna, M.; Banks, E.; Sivachenko, A.; Cibulskis, K.; Kernytsky, A.; Garimella, K.; Altshuler, D.; Gabriel, S.; Daly, M.; et al. The Genome Analysis Toolkit: A MapReduce Framework for Analyzing next-Generation DNA Sequencing Data. *Genome Res.* **2010**, *20*, 1297–1303, doi:10.1101/gr.107524.110.

12. Ramírez, F.; Ryan, D.P.; Grüning, B.; Bhardwaj, V.; Kilpert, F.; Richter, A.S.; Heyne, S.; Dündar, F.; Manke, T. deepTools2: A next Generation Web Server for Deep-Sequencing Data Analysis. *Nucleic Acids Res.* **2016**, *44*, W160-165, doi:10.1093/nar/gkw257.

13. Zhang, Y.; Liu, T.; Meyer, C.A.; Eeckhoute, J.; Johnson, D.S.; Bernstein, B.E.; Nusbaum, C.; Myers, R.M.; Brown, M.; Li, W.; et al. Model-Based Analysis of ChIP-Seq (MACS). *Genome Biol.* **2008**, *9*, R137, doi:10.1186/gb-2008-9-9-r137.

14. Zhu, L.J.; Gazin, C.; Lawson, N.D.; Pagès, H.; Lin, S.M.; Lapointe, D.S.; Green, M.R. ChIPpeakAnno: A Bioconductor Package to Annotate ChIP-Seq and ChIP-Chip Data. *BMC Bioinformatics* **2010**, *11*, 237, doi:10.1186/1471-2105-11-237.

15. Yu, G.; He, Q.-Y. ReactomePA: An R/Bioconductor Package for Reactome Pathway Analysis and Visualization. *Mol. Biosyst.* **2016**, *12*, 477–479, doi:10.1039/C5MB00663E.
